# Identification of *SLC22A17* DNA methylation hotspot as a potential biomarker in cutaneous melanoma

**DOI:** 10.1186/s12967-024-05622-9

**Published:** 2024-10-02

**Authors:** Alessandro Lavoro, Luca Falzone, Giuseppe Gattuso, Giuseppe N. Conti, Rosario Caltabiano, Gabriele Madonna, Mariaelena Capone, James A. McCubrey, Paolo A. Ascierto, Massimo Libra, Saverio Candido

**Affiliations:** 1 Department of Biomedical and Biotechnological Sciences, University of Catania, Catania, I-95123 Italy; 2 Department of Medical and Surgical Sciences and Advanced Technologies “G.F. Ingrassia”, University of Catania, Catania, I-95123 Italy; 3 Melanoma Cancer Immunotherapy and Innovative Therapy Unit, Istituto Nazionale Tumori IRCCS Fondazione G. Pascale, Napoli, I-80131 Italy; 4 Department of Microbiology and Immunology, Brody School of Medicine, East Carolina University, Greenville, NC 27858 USA; 5 Research Center for Prevention, Diagnosis and Treatment of Cancer, University of Catania, Catania, I- 95123 Italy

**Keywords:** Epigenetics, DNA methylation, Tumor microenvironment, *SLC22A17*, 5-Azacytidine, Cancer biomarkers, Cutaneous melanoma, Bioinformatics

## Abstract

**Background:**

Cancer onset and progression are driven by genetic and epigenetic alterations leading to oncogene activation and the silencing of tumor suppressor genes. Among epigenetic mechanisms, DNA methylation (methDNA) is gaining growing interest in cancer. Promoter hypomethylation is associated with oncogene activation while intragenic methDNA can be involved in transcriptional elongation, alternative spicing, and the activation of cryptic start sites. Several genes involved in the modulation of the tumor microenvironment are regulated by methDNA, including the *Solute Carrier Family 22 Member 1*7 (*SLC22A17*), which is involved in iron trafficking and extracellular matrix remodeling cooperating with the *Gelatinase-Associated Lipocalin* (*NGAL*) ligand. However, the exact role of intragenic methDNA in cancer has not been fully investigated. Therefore, the aim of the present study is to explore the role of methDNA in the regulation of *SLC22A17* in cutaneous melanoma (CM), used as a tumor model.

**Methods:**

Correlation and differential analyses between *SLC22A17* expression and methDNA were performed using the data contained in The Cancer Genome Atlas and Gene Expression Omnibus databases. Functional studies on melanoma cell lines treated with 5-Azacytidine (5-Aza) were conducted to assess the correlation between methDNA and *SLC22A17* expression. A validation study on the diagnostic potential of the in silico-identified *SLC22A17* methDNA hotspot was finally performed by analyzing tissue samples obtained from CM patients and healthy controls.

**Results:**

The computational analyses revealed that *SLC22A17* was significantly downregulated in CM, and its expression was related to promoter hypomethylation and intragenic hypermethylation. Moreover, *SLC22A17* overexpression and hypermethylation of two intragenic methDNA hotspots were associated with a better clinical outcome in CM patients. The correlation between *SLC22A17* methDNA and expression was confirmed in 5-Aza-treated cells. In agreement with in silico analyses, the *SLC22A17* promoter methylation hotspot showed higher methDNA levels in CM samples compared to nevi. In addition, the methDNA levels of this hotspot were positively correlated with advanced CM.

**Conclusions:**

The *SLC22A17* methDNA hotspot could represent a promising biomarker for CM, highlighting the regulatory role of methDNA on *SLC22A17* expression. These results pave the way for the identification of novel epigenetic biomarkers and therapeutic targets for the management of CM patients.

**Supplementary Information:**

The online version contains supplementary material available at 10.1186/s12967-024-05622-9.

## Introduction

DNA methylation (methDNA), one of the most characterized epigenetic mechanisms, plays a critical role in the alteration of gene expression and the activation of molecular pathways involved in carcinogenesis, progression, and drug resistance [1–5]. This epigenetic regulatory mechanism consists of the transfer of a methyl group (-CH_3_) from S-adenosyl-L-methionine (SAM) to the carbon-5 position of the cytosine base within the cytosine-guanine (CpG) dinucleotide, forming 5-methylcytosine (5mC) [6]. Promoter methDNA is negatively associated with gene expression since the hypermethylation of CpG islands, mainly located in the proximal promoter region, prevents the binding of transcription factors to their relative consensus sequences [7–12]. Intragenic methDNA also plays a role in the regulation of gene expression by mediating transcriptional elongation efficiency, as well as alternative splicing, transposable element regulation, and the activation of cryptic start sites; however, the function and significance of gene body methDNA in the regulation of gene expression should be further investigated [13–17].

Recent studies highlight the relationship between methDNA status and the aberrant expression of key genes involved in the regulation of the tumor microenvironment (TME). For instance, it has been demonstrated that methDNA modulates the behavior of fibroblasts, immune cells, and stromal cells, promoting a pro-tumoral phenotype [18–22]. Among TME components, the degradation of extracellular Matrix (ECM) plays a pivotal role in carcinogenesis, and its alteration is closely related to the aberrant expression of Matrix Metalloproteinases (MMPs), including *MMP-2* and *MMP-9* [23]. In this context, our previous studies showed that the activation and protein stability of MMP-9 are enhanced by the interaction with Neutrophil Gelatinase Associated Lipocalin (NGAL), also known as Lipocalin 2 (LCN2), thereby improving the MMP-9 gelatinolytic activity that leads to ECM degradation, tumoral invasiveness, and metastatic process [24, 25]. Besides ECM degradation, iron metabolism is also critical for cancer cell proliferation and survival, whose trafficking is regulated by the NGAL receptor (*NGALR*), better known as *Solute Carrier Family 22 Member 17* (*SLC22A17*) [26]. Mechanistically, the iron-siderophore cellular influx and efflux systems are mediated by *NGAL-SLC22A17* complex internalization and recycling, increasing cellular iron uptake and depletion, respectively. The SLC22A17 isoforms 1 and 2 have been described as bilateral iron transporters, while isoform 3 seems to mediate cellular iron influx exclusively [27, 28]. Considering the involvement of SLC22A17 in iron trafficking, the dysregulation of *SLC22A17* plays a key role in the development, progression, and drug resistance of several tumors, including cutaneous melanoma (CM) [29–32]. However, no relevant mutations or Single Nucleotide Polymorphisms (SNPs) affecting the aberrant expression of *SLC22A17* in cancer have been described, nor the epigenetic alterations associated with *SLC22A17* dysregulation in cancer.

Since the current literature lacks evidence on the mechanisms responsible for *SLC22A17* dysregulation in melanoma, in the present study, we investigated the role of methDNA in the regulation of *SLC22A17* in CM. The diagnostic and prognostic potential of this novel epigenetic biomarker was also explored in CM patients. The identification of genetic and epigenetic factors involved in CM progression and relapse is also essential to develop effective therapeutic options for the management of CM patients.

## Materials and methods

### Omics data collection

The differential analysis of *SLC22A17* expression and methDNA status between CM samples and normal tissues (nevi) was performed by analyzing the GSE112509 and GSE120878 datasets, respectively (https://www.ncbi.nlm.nih.gov/geo/, accessed on January 2023). The GEO datasets were selected according to the following criteria: (i) CM and nevi samples had to be included in the same dataset; (ii) the sample size had to be greater than 50 samples for expression and methDNA datasets; (iii) the data contained in the methDNA dataset had to be generated by the Illumina HumanMethylation450 BeadChip array platform. The GSE112509 dataset included the expression data (DESeq2 normalized counts - Illumina HiSeq 2000) of 57 primary CM samples and 23 melanocytic nevi, while the GSE120878 dataset contained methDNA levels (Beta value - Illumina Infinium HumanMethylation450 BeadChip array) of 89 primary invasive CM and 73 nevi. Correlation analysis between *SLC22A17* expression and methDNA was not executed in GEO datasets since the CM and nevi samples belonged to two different cohorts.

The gene expression RNAseq - TOIL RSEM FPKM and transcript expression RNAseq - TOIL RSEM FPKM datasets, as well as the methDNA - DNA methylation (Methylation450K) dataset, were used to perform gene/transcript expression and methDNA profiling of *SLC22A17* in melanoma (SKCM) samples (*N* = 470) included in the TCGA Pan-Cancer cohort. Correlation analysis was also performed in TCGA SKCM cohort between *SLC22A17* gene/transcript expression and methDNA data that are both available in TCGA database. Both expression values and methDNA levels of *SLC22A17* were retrieved using UCSC Xena Functional Genomics Explorer (https://xenabrowser.net/, accessed in January 2023). Differential analysis of *SLC22A17* expression and methDNA levels was not performed in TCGA SKCM cohort since only two normal samples are available in this cohort.

### Melanoma patients and healthy controls

A group of 37 CM patients (age range 30–88 years; average age 58.78 years) and 15 nevi samples with no atypical histological features (age range 18–55 years; average age 37.13) was enrolled at the National Cancer Institute “Fondazione G. Pascale”, Naples (Italy). The patients and controls came from the same geographic region and had the same ethnic background. Formalin-fixed paraffin-embedded (FFPE) tissue samples were obtained from both patients and controls using standard procedures. The FFPE tissues were collected from April 2020 to July 2020 at the Melanoma Cancer Immunotherapy and Innovative Therapy Unit of the National Cancer Institute ‘Fondazione ‘G. Pascale’ (Naples, Italy), retrospectively retrieving the FFPE samples from the archives of the Pathology Unit (years 2008–2020). The study was conducted in accordance with the principles of the Declaration of Helsinki and was approved by the Institutional Review Board of the National Cancer Institute “Fondazione G. Pascale” (Naples, Italy) (protocol n. 33/17 and 37/22 oss). Both CM patients and controls involved in the study provided informed consent. The sociodemographic and clinical characteristics of all the CM patients and healthy individuals are reported in Table 1.

**Table 1:** Sociodemographic and clinical characteristics of CM patients and healthy controls

	Melanoma		Nevi	
	*N*.	(%)	*N*.	(%)
**Samples**	37	(71.15%)	15	(28.85%)
**Age (years)**				
< 45	8	(21.62%)	10	(66.67%)
45 − 60	12	(32.43%)	5	(33.33%)
> 60	17	(45.95%)	0	(0%)
Chi-square test		*p* = 0.0014		
**Gender**				
Male	22	(59.46%)	5	(33.33%)
Female	15	(40.54%)	10	(66.67%)
Fisher’s exact test		*p* = 0.1274		
**Stage**				
pT1	1	(2.70%)		
pT2	4	(10.81%)		
pT3	14	(37.84%)		
pT4	16	(43.24%)		
Missing	2	(5.41%)		
**Breslow (mm)**				
< 2	4	(10.81%)		
2–4	15	(40.54%)		
> 4	15	(40.54%)		
Missing	3	(8.11%)		
**Clark level**				
III - IV	6	(16.22%)		
IV - V	27	(72.97%)		
Missing	4	(10.81%)		
**BRAF Status**				
Wild type	8	(21.62%)		
Mutated	14	(37.84%)		
Missing	15	(40.54%)		
**Number of Mitosis**				
< 2	5	(13.51%)		
2–4	13	(35.14%)		
> 4	15	(40.54%)		
Missing	4	(10.81%)		
**TILs**				
Absent	3	(8.11%)		
No Brisk	18	(48.65%)		
Brisk	11	(29.73%)		
Missing	5	(13.51%)		
**Vascular Invasion**				
Negative	30	(81.08%)		
Positive	4	(10.81%)		
Missing	3	(8.11%)		
**Ulceration**				
Negative	12	(32.43%)		
Positive	23	(62.16%)		
Missing	2	(5.41%)		

### Cell cultures and treatment

The A375 (Cat. No. CRL-1619), A2058 (Cat. No. CRL-3601), and MeWo (Cat. No. HTB-65) melanoma cell lines were obtained from the American Type Culture Collection (ATCC) (Rockville, MD, USA), the M14 and SK-MEL-28 cell lines were already available at the cell biobank of the Experimental Oncology Laboratory (Department of Biomedical and Biotechnological Sciences, University of Catania), whereas the SK-MEL-23 and WM115 cells were available at the National Cancer Institute G. Pascale of Naples. A375, A2058, M14, SK-MEL-23, SK-MEL-28, and WM115 were cultured in a complete RPMI-1640 medium (Cat. No. 10-040-CV - Corning^®^ Life Sciences), while MeWo cells were cultured in a complete EMEM medium (Cat. No. 15-010-CV - Corning^®^ Life Sciences), both supplemented with 10% Fetal Bovine Serum (FBS) (Cat. No. 35-079-CV), 2 mmol/L of L-glutamine (Cat. No. 25-005-CI), 100 UI of penicillin and 100 µg/mL streptomycin (Cat. No. 30-001-CI), all provided by Corning^®^ Life Sciences. Each cell line was seeded in 100 mm cell-culture dishes (Cat. No. 0030702115, Eppendorf) at a density of 1 × 10^6^ cells and grown in a humidified incubator at 37 °C and 5% CO_2_. Cell pellets were collected by scraping cell cultures in cold PBS 1X (Cat. No. 21-040-CV - Corning^®^ Life Sciences) and frozen at -80 °C until analyses.

A375, SK-MEL-23, SK-MEL-28, and WM115 cell lines were also treated for 5 days with the demethylating agent 5-Azacytidine (5-Aza) (Cat. No. A2385, Sigma-Aldrich, Darmstadt, Germany). Specifically, A375 and WM115 cells were treated at a concentration of 0.75 µM, SK-MEL-23 at 1.9 µM, and SK-MEL-28 cells at 3.5 µM according to the IC_50_, which was computed by treating each cell line with different doses of 5-Aza (Supplementary Fig. S1). To this end, all CM cell lines were seeded in 96-well plates at a density of 4 × 10^3^ cells per well in 100 µL of complete medium, except for A375 (2 × 10^3^ per well), prior to the treatment with serial dilutions of 5-Aza (100 − 10–1 − 0.1–0.01 − 0.001 µM). DMSO (Cat. No. D8418 – Sigma Aldrich) was used as a vehicle control for all treatments. After 72 h of treatment, the culture medium was replaced with 100 µL of fresh medium supplemented with MTT (Cat. No. 158990010 - Thermo Fisher Scientific™, Waltham, MA, United States) solution (5 mg/mL in PBS 1X) at a final concentration of 0.5 mg/mL and the plate was incubated at 37 °C and 5% CO_2_ for 3 h. Following the incubation, the MTT solution was removed, and 100 µL of DMSO was added to each well to dissolve formazan crystals. Finally, the absorbance of each well was measured at 620 nm using the Tecan Sunrise™ microplate reader (TECAN, Schweiz ACT, Switzerland) to retrieve the optical density (OD) values of each well. All experiments were performed in duplicate.

### Genomic DNA and total RNA extraction

Genomic DNA from each CM cell line was extracted using the PureLink ™ Genomic Mini Kit (Cat. No. K1820-01 - Invitrogen, Thermo Fisher Scientific) according to the manufacturer’s instructions. For the extraction of genomic DNA from FFPE tissues (four sections with a thickness of 8 μm), the deparaffinization solution (Cat. No. 19093 - Qiagen GmbH) and the QIAamp DNA FFPE Tissue Kit (Cat. No. 56404 – Qiagen GmbH) were used according to the manufacturer’s protocols. Total RNA was extracted from each melanoma cell line using the PureLink^®^ RNA Mini Kit (Cat. No. 12183018 A – Invitrogen, Thermo Fisher Scientific™, Waltham, MA, United States) according to the manufacturer’s instructions. Nanodrop-1000 was used to assess the quantity and quality of all extracted DNA and RNA samples. Following the extraction procedures, genomic DNA samples were frozen at -20 °C, while RNA samples were frozen at -80 °C until analyses.

### Bisulfite conversion and Sanger sequencing

The *SLC22A17* methDNA profile of each CM cell line was obtained by bisulfite conversion, followed by PCR amplification and Sanger sequencing. Briefly, 1,200 ng of genomic DNA were bisulfite-converted using the EpiTect Plus DNA Bisulfite Kit (Cat. No. 59124 – Qiagen GmbH) according to the manufacturer’s protocol. Then, the amplification of bisulfite-converted samples was executed by preparing a reaction mix containing 100 ng of the bisulfite-converted DNA, 10 µL of the 2X ddPCR Supermix for Probes (No dUTP) (Cat. No. 1863024 - Bio-Rad Laboratories Inc, Hercules, CA, United States), 10 µM (final concentration) of forward and reverse primers for each target, and molecular biology grade H_2_O to a final volume of 20 µL. PCR thermal conditions and primer sequences are reported in Table 2. The Bisulfite Primer Seeker tool (https://www.zymoresearch.eu/pages/bisulfite-primer-seeker - accessed in March 2023) was used to design the bisulfite primers. The primer localizations for each PCR target are shown in Supplementary Fig. S2.

**Table 2:** Primers and amplification conditions

Name	Sequence	Ampl. conditions
*SLC22A17* amplification from bisulfite-converted DNA		
Prom1 Fw	5’-TTAGGGTTTAGGGGAGGGAG-3’	95 °C for 10 min, followed by 40 cycles of 94 °C for 30 s, 55 °C for 1 min, and finally 98 °C for 10 min
Prom1 Rev	5’-CTACCTAAACTAACTACTATCCTTCAA-3’	
Prom2 Fw	5’-GTGATTTTTATAGTGTTGTYGATTTT-3’	
Prom2 Rev	5’-CTACAAAACTACAAAACRAAATCTCTTC-3’	
Prom3 Fw	5’-GTGAGTATAGGAAGGTTATTATAGTTTT-3’	
Prom3 Rev	5’-TAACTAAAAACAACCTCCCAATAC-3’	
Prom4 Fw	5’-ATATTAGATTTTATTGGGGATGTGAGAA-3’	
Prom4 Rev	5’-AAAACTATAATAACCTTCCTATACTCAC-3’	
Body1 Fw	5’-GGATTTTTAGGGTTTTGAGATTTTTTTA-3’	
Body1 Rev	5’-AATCAATAATAAAAATAACCAAAATCAA-3’	
Body2 Fw	5’-TTTGTTTAGGTTTTTTTGAAGAATTTAG-3’	
Body2 Rev	5’-TACTATCAAAAAAATAACACCTTATTCC-3’	
Body3 Fw	5’-GGGTTAGGTTAGTAGTTGGAAT-3’	
Body3 Rev	5’-ACRAATAACATAAACAATAAAACTATAAAA-3’	
3’UTR1 Fw	5’-GTTATAGYGGGTAGGGGGTG-3’	
3’UTR1 Rev	5’-AACCTAAACCTAATCATAACTCTAAAAA-3’	
3’UTR2 Fw	5’-GTTATAGYGGGTAGGGGGTG-3’	
3’UTR2 Rev	5’-ATTCTCAACATTATACTACTACCRAA-3’	
***SLC22A17 *** **MSRE-qPCR**		
MSRE UpP Fw	5’-AAGGATGCGCTGTCCTCTG-3’	50 °C for 2 min and 95 °C for 10 min, followed by 40 cycles of 95 °C for 10 s, 60 °C for 30 s, and 72 °C for 30 s
MSRE UpP Rev	5’-AGAGCGGGATCTCTTCGAGC-3’	
MSRE DownP Fw	5’-GAGGCAATGGTTGAAGTCCG-3’	
MSRE DownP Rev	5’-CTAATGCCTCTGGCTGGGAG-3’	
MSRE Body Fw	5’-AGCAACGAACAGAGCCTGAA-3’	
MSRE Body Rev	5’-ATCCTGGGCTTCACCAAGTG-3’	
***SLC22A17 *** **RT-qPCR**		
All var Fw	5’-TGGTTTGTTCCTGGAGTCCG-3’	50 °C for 2 min and 95 °C for 10 min, followed by 40 cycles of 95 °C for 10 s, 60 °C for 30 s, and 72 °C for 30 s
All var Rev	5’-GCATGGGCAATGAAGTTGGT-3’	
Var1 Fw	5’-GGTCACCGTGGACCGATTT-3’	
Var1 Rev	5’-TTGGGGTTCCCTTGTTGAGC-3’	
Var2 Fw	5’-ATTGGCGATTCCTACAGCGA-3’	
Var2 Rev	5’-AGCCTCGTTCAGATAATCCCAC-3’	
Var3 Fw	5’-GGTGTCTACCTGATGCCGAAT-3’	
Var3 Rev	5’-CGGTTTCGCTCAGCCAGGAT-3’	
*GAPDH* Fw	5’-AGAAGGCTGGGGCTCATTTG-3’	
*GAPDH* Rev	5’-AGGGGCCATCCACAGTCTTC-3’	
***SLC22A17 *** **MSRE-ddPCR**		
MSRE DownP Fw	5’-GAGGCAATGGTTGAAGTCCG-3’	95 °C for 10 min, followed by 40 cycles of 94 °C for 30 s, 55 °C for 1 min, and finally 98 °C for 10 min (ramp rate 2 °C/s)
MSRE DownP Rev	5’-CTAATGCCTCTGGCTGGGAG-3’	
MSRE DownP probe	[FAM]5’–GCCGCTGCACGAGGGGTCGG-3’[BHQ1]	
methCTRL Fw	5’–CACTATAGGGAGACCCAAG-3’	
methCTRL Rev	5’-AACTTGTGGCCGTTTAC-3’	
methCTRL probe	[HEX]5’–CTGTTCACCGGGGTGG-3’[BHQ1]	

Following amplification, PCR products were cleaned up using the PureLink PCR Purification Kit (Cat. No. K310001 - Thermo Fisher Scientific Inc., Waltham, MA, United States) and sequenced with the Mix2Seq Kit (Eurofins Genomics Germany GmbH, Ebersberg, Germany) according to the manufacturer’s protocols. Chromas Lite software version 2.6.6 (https://technelysium.com.au/wp/chromas/) (accessed in June 2023) was used to analyze the DNA sequences.

### MSRE-qPCR

The methylation-sensitive restriction enzyme (MSRE) assay was performed for each DNA sample derived from both untreated and 5-Aza-treated CM cell lines. In particular, three different reaction tubes (final volume 10 µL) were prepared by mixing 200 ng of genomic DNA, 1X CutSmart Buffer (Cat. No. B7204), and 20 UI of HpaII enzyme (Cat. No. R0171S) for tube 1 (Mix HpaII), 20 UI of MspI enzyme (Cat. No. R0106S) for tube 2 (Mix MspI), and no enzyme for tube 3 (Mix no enzyme) (all the reagents were purchased from New England Biolabs, Germany). The reaction tubes were incubated at 37 °C for 1 h, and enzyme restriction was stopped with 1 mg/mL of Proteinase K (Cat. No. EO0491 - Thermo Fisher Scientific Inc., Waltham, MA, United States), incubating the samples at 55 °C for 30 min and 95 °C for 10 min. After the standard MSRE digestion, 20 ng of each digested sample was used for downstream SYBR green-based qPCR amplification to assess the methDNA levels of three *SLC22A17* methDNA hotspots mapped in the upstream promoter (chr14:23,821,982 − 23,822,182 - GRCh37/hg19), downstream promoter (chr14:23,821,211 − 23,821,359 - GRCh37/hg19), and body (chr14:23,816,960 − 23,817,116 - GRCh37/hg19) regions to obtain a representative estimation of their methDNA status. The upstream promoter hotspot included two HpaII recognition sites and the cg01557297, the downstream promoter hotspot contained one HpaII site and the cg17199325 with overlapping position, while one HpaII site and the cg10130460 probeset were included in the selected body region (Supplementary Fig. S2). Briefly, the amplification mix was prepared as follows: 10 µL of Luminaris Color HiGreen qPCR Master Mix, high ROX (Cat. No. K0361 – Invitrogen, Thermo Scientific™, Waltham, MA, United States), 10 µM (final concentration) of forward e reverse primers for each target, 1 µL of MSRE-digested DNA (20 ng/µL) and molecular biology grade H_2_O up to a final volume of 20 µL. PCR thermal conditions and primer sequences are reported in Table 2. The amplification signals were measured using the AB 7300 Real-Time PCR System (Applied Biosystems; Thermo Fisher Scientific, Inc.), and the methDNA percentage of each target was computed using the formula:

$$\:100\times\:{2}^{-(\text{C}\text{t}\:\text{o}\text{f}\:\text{u}\text{n}\text{d}\text{i}\text{g}\text{e}\text{s}\text{t}\text{e}\text{d}\:\text{s}\text{a}\text{m}\text{p}\text{l}\text{e}\:-\:\text{C}\text{t}\:\text{o}\text{f}\:\text{H}\text{p}\text{a}\text{I}\text{I}\:\text{d}\text{i}\text{g}\text{e}\text{s}\text{t}\text{e}\text{d}\:\text{s}\text{a}\text{m}\text{p}\text{l}\text{e})}$$

The efficiency of MSRE digestion was evaluated by considering the amplification signal (cutoff Ct: > 35) of each methDNA target in MspI mixes.

### RT-qPCR

The analysis of *SLC22A17* expression levels in CM cell lines was performed considering the coding RefSeq sequences available on the NCBI database. The oligos for RT-qPCR were designed to selectively amplify the NM_020372.4 (Transcript variant 1: Var 1), NM_016609.7 (Transcript variant 2: Var 2), and NM_001289050.1 (Transcript variant 3: Var 3), as well as to detect the expression of all transcripts together (All Vars).

Reverse transcription was carried out on each RNA sample obtained from untreated and 5-Aza-treated cell lines using SuperScript IV Reverse Transcriptase (Cat. No. 18090010, Invitrogen, Thermo Fisher Scientific™, Waltham, MA, United States). Briefly, the reaction mix was prepared by mixing 500 ng of total RNA, 1 µL of random hexamers (100 µM final concentration), 1 µL of Deoxynucleotide Triphosphates (dNTPs) (100 µM final concentration), and DEPC-treated water up to a final volume of 14 µL. The samples were then incubated at 65 °C for 5 min and on ice for 1 min. Following the incubation, 6 µL of the RT reaction, containing 4 µL of 5x SSIV Buffer, 1 µL of DTT (100 mM final concentration), and 1 µL of SuperScript IV Reverse Transcriptase (200 UI/µL), was added to each sample. Finally, the samples were incubated at 23 °C for 10 min, 55 °C for 10 min, and 80 °C for 10 min.

The amplification of cDNA was performed with the AB 7300 Real-Time PCR System (Applied Biosystems; Thermo Fisher Scientific, Inc.) by preparing a reaction mix containing 10 µL of Luminaris Color HiGreen qPCR Master Mix, high ROX (Cat. No. K0361 – Invitrogen, Thermo Scientific™, Waltham, MA, United States), 10 µM (final concentration) of forward and reverse primers for each target, 1 µL of cDNA (25 ng/µL), and molecular biology grade H_2_O up to a final volume of 20 µL. Primers and amplification conditions are reported in Table 2. The ΔΔCt relative quantification method [33] was performed to quantify the expression levels of *SLC22A17* All Vars, as well as Var 1, Var 2, and Var 3, using *Glyceraldehyde-3-Phosphate Dehydrogenase* (*GAPDH*) signal value as the control reference. All experiments were performed in duplicate.

### MSRE-ddPCR

The analysis of the in silico identified *SLC22A17* methDNA hotspot (cg17199325), belonging to the downstream promoter region, was performed on CM and nevi FFPE tissues using the custom method Methylation-Sensitive Restriction Enzyme – droplet digital PCR (MSRE-ddPCR) as previously described [34]. Briefly, two different amplification mixes were prepared for each sample, one containing HpaII restriction enzyme (Cat. No. R0171S - New England Biolabs, Germany) and one without enzyme as an undigested control. In particular, each amplification mix (final volume 22 µL) was prepared using 11 µL of 2X ddPCR Supermix for Probes (No dUTP) (Cat. No. 1863024 - Bio-Rad Laboratories Inc, Hercules, CA, United States), 10 UI of HpaII in each HpaII mix, no enzyme in the undigested control mix, 900 nM of forward and reverse primers, and 450 nM of probes for the *SLC22A17* target (FAM) and methylation CTRL (methCTRL) (HEX). MethCTRL consists of an exogenous sequence derived from the fluorescent protein Clover, which contains one unmethylated CCGG restriction site to evaluate the digestion efficiency of HpaII in the MSRE-ddPCR reaction. Probe and primer sequences, as well as amplification conditions, are reported in Table 2. Up to 5 µL of DNA sample (∼ 20 ng) and 10^− 6^ ng of methCTRL were added to each MSRE-ddPCR mix and incubated at 37 °C for 30 min before droplet generation by QX100™ Droplet Generator and PCR amplification by the C1000 Thermal Cycler (Bio-Rad Laboratories Inc, Hercules, CA, United States). Finally, the QX200™ Droplet Reader (Bio-Rad Laboratories Inc, Hercules, CA, United States) and QuantaSoft software, version 1.7.4 (QuantaSoft, Prague, Czechia) were used for the absolute quantification (copies/µL) of the *SLC22A17* methDNA target. Amplitude thresholds were set manually by the operator based on positive and negative droplet amplitudes. The *SLC22A17* methDNA percentage was calculated considering the ratio between the amplification signals in HpaII and undigested control mixes for each sample. The methDNA percentage of *SLC22A17* hotspot was normalized using the enzymatic digestion coefficient obtained from the methCTRL analysis.

### Statistical analyses

The differential analysis of *SLC22A17* expression and methDNA data, retrieved from publicly available datasets, was performed using the Mann-Whitney test for comparing two groups, while the comparison analyses of more than two groups were executed using the Kruskal-Wallis test and Dunn’s multiple comparisons test with GraphPad Prism (version 8.0.2) (GraphPad Software, San Diego, CA, USA) since all expression and methDNA groups did not pass the Shapiro-Wilk normality test. The difference among the comparison groups was reported as Fold Change (FC), computed using the formula: $\:{\pm\:2}^{|median\:1-median\:2|}$. The methDNA difference between the comparison groups was evaluated by the difference in Beta values for each CG probeset. Correlation analysis between *SLC22A17* gene/transcript expression and CG methDNA levels in TCGA SKCM samples was conducted using Pearson’s correlation test. Overall survival (OS) and Progression Free Interval (PFI) analyses were performed by Kaplan-Meier analysis on GraphPad Prism, stratifying TCGA SKCM samples into two groups according to *SLC22A17* gene/transcript expression and methDNA levels (above and below the median values). Chi-square and p-values were estimated using the Log-rank (Mantel-Cox) test, and the median survival time was also calculated for each Kaplan-Meier curve.

For in vitro experiments, statistical analyses of *SLC22A17* expression levels (All Vars, Var 1, Var 2, and Var 3) were performed using a two-tailed T-test for comparing two groups, whereas the one-way ANOVA multiple comparisons test was used to compare more than two groups. Differential analysis of methDNA levels was conducted by the Mann-Whitney test and the Kruskal-Wallis test and Dunn’s multiple comparisons test. The 5-Aza IC_50_ values were calculated for each melanoma cell line using GraphPad Prism.

Regarding the validation study on FFPE samples, the distribution of CM patients and healthy controls according to sociodemographic and clinical characteristics was reported as absolute numbers and corresponding percentages. The heterogeneity of the sample groups (age and gender) was tested by the Chi-square (and Fisher’s exact) test using GraphPad Prism. The statistical analysis of the *SLC22A17* downstream promoter hotspot was performed using the Mann-Whitney test and the Kruskal-Wallis test and Dunn’s multiple comparisons test. To evaluate the sensitivity and specificity of the diagnostic test, the Receiver Operating Characteristic (ROC) analysis was also executed using GraphPad Prism.

## Results

### In silico analyses of *SLC22A17* expression and methDNA in CM and nevi samples

**Fig. 1:**
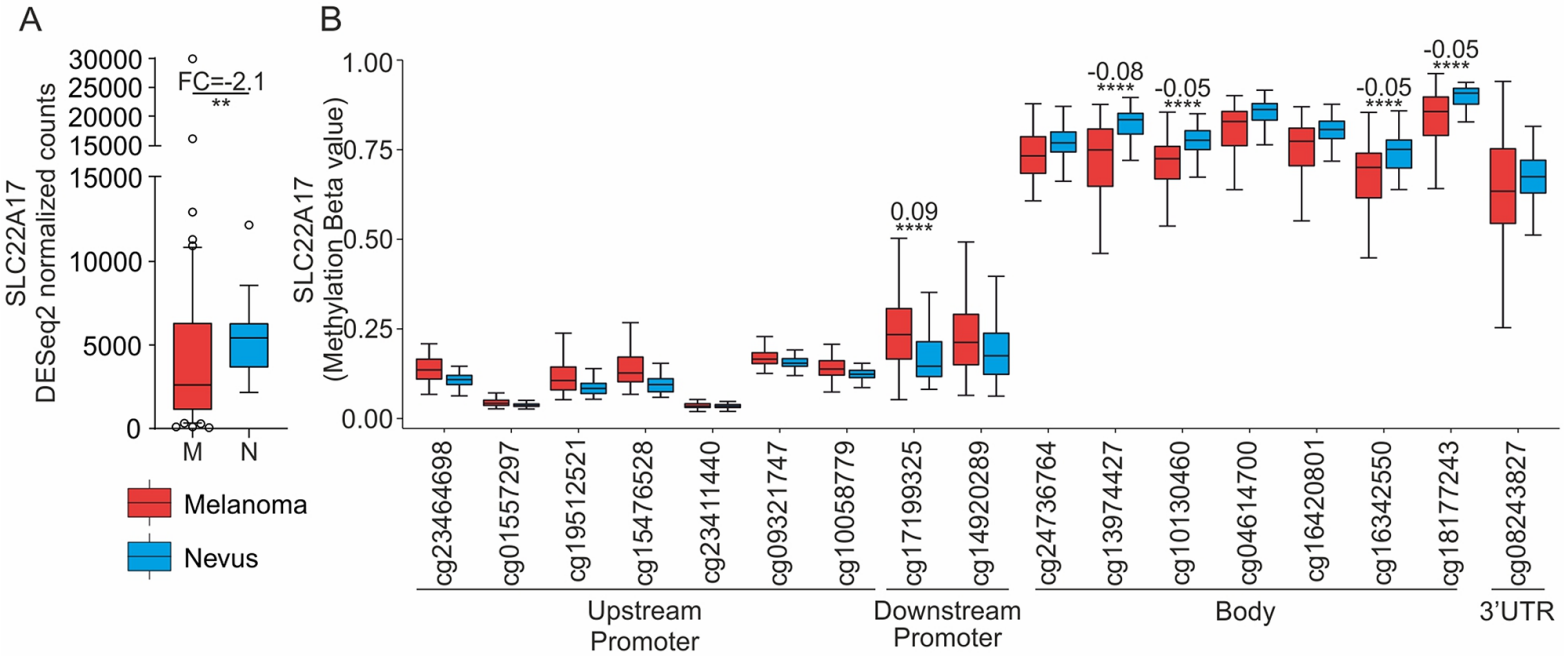
*SLC22A17* gene expression and methDNA analyses between CM and nevi samples in GEO datasets. (**A**) The differential analysis of gene expression between CM and nevi samples was performed using the dataset GSE112509. The difference between the comparison groups is reported as the median values ratio of CM versus nevi samples. Statistical significance was evaluated using the Mann-Whitney test. (**B**) Differential analysis of methDNA levels between CM and nevi was performed using the GSE120878 dataset. Beta difference values were calculated as the differences between the medians of each group, and the Mann-Whitney test was used to calculate the statistical significance. Red and blue boxes indicate CM and nevi samples, respectively. *p-value*: ** < 0.01, **** < 0.0001

As previously stated, the *NGAL/SLC22A17/MMP-9* network plays a critical role in the TME remodeling. Although the epigenetic regulation of *NGAL* and *MMP-9* in cancer was widely investigated, no data on the role of methDNA in the regulation of *SLC22A17* in CM are reported in the literature. On these bases, in silico differential analyses were performed comparing the gene expression of *SLC22A17* between CM and nevi biopsies retrieved from the GSE112509 dataset. The obtained results indicated that *SLC22A17* was significantly downregulated in CM samples compared to nevi (FC = -2.1, *p* < 0.01) (Fig. 1A). Moreover, the GSE120878 dataset was used to evaluate *SLC22A17* methDNA profiling, revealing that all the CG probesets of the upstream and downstream promoter regions were collectively hypomethylated (median Beta values < 0.25) in both CM samples and nevi tissues, whereas the CG probesets of the body and 3’UTR regions were methylated in both groups (median Beta values > 0.6). Notably, the differential analysis showed that methDNA levels of CG probesets within the upstream promoter region were weakly higher in CM samples compared to nevi; however, no statistical significance was achieved. A similar trend was observed for the CG probesets belonging to the downstream promoter region (cg17199325 and cg14920289), which showed higher methDNA levels in CM compared to nevi, reaching statistical significance only for the cg17199325 probeset (Beta difference = 0.09, *p* ≤ 0.0001). Conversely, an opposite trend was observed for body and 3’UTR CG probesets, whose methDNA levels were higher in nevi compared to CM samples. Notably, only four out of seven body CG probesets (cg13974427, cg10130460, cg16342550, and cg18177243) were significantly differentially methylated (Beta difference ranged from − 0.05 to -0.08, *p* < 0.0001) (Fig. 1B).

**Fig. 2:**
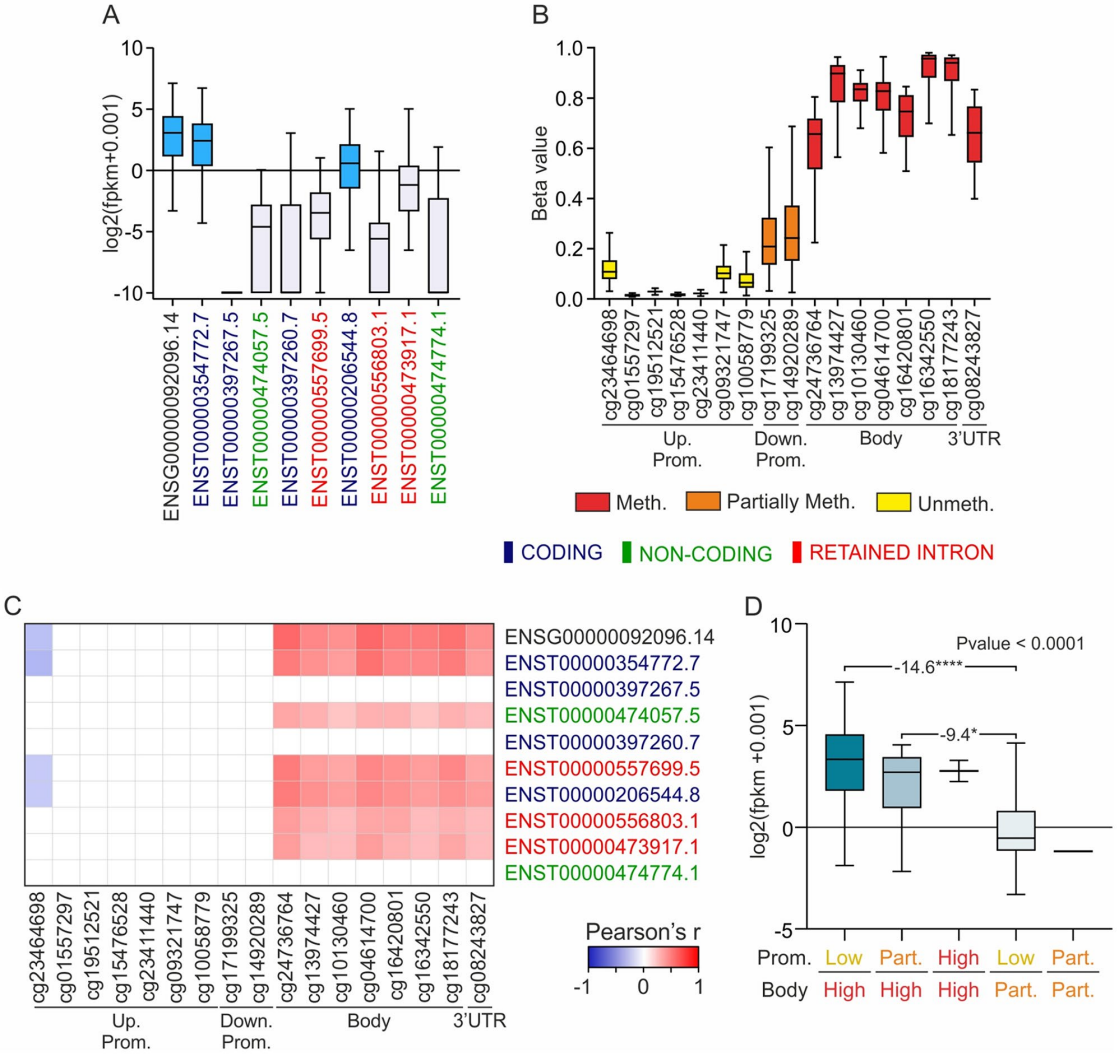
Profiling and correlation analyses of *SLC22A17* gene/transcript expression and methDNA in the TCGA SKCM cohort. (**A**) Box plot analysis of gene and transcript expression of *SLC22A17* in the TCGA Pan-Cancer SKCM cohort using TOIL RSEM FPKM normalized data. The cyan boxes indicate that the median gene/transcript expression levels are positive. (**B**) methDNA profiling of all the *SLC22A17* CG probesets retrieved from the Methylation450K dataset. Red, orange, and yellow boxes indicate hypermethylated (Beta value > 0.6), partially methylated (0.2 ≤ Beta value  ≤ 0.6), and unmethylated (Beta value < 0.2) CG probesets, respectively. (**C**) Heatmap of correlation analysis between *SLC22A17* gene/transcript expression and CG probesets methDNA levels in TCGA Pan-Cancer SKCM samples. (adapted from Heatmapper tool available at http://www.heatmapper.ca/expression/). The positive correlation pairs are reported in red (Pearson’s *R* ≥ 0.2, *p* ≤ 0.05), while negative correlations are indicated in blue (Pearson’s *R* ≤ -0.2, *p* ≤ 0.05). The coding transcripts of *SLC22A17* are labeled in blue, the non-coding transcripts in green, and red is used for transcripts with retained introns. (**D**) Differential analysis of *SLC22A17* gene expression was performed by stratifying the SKCM samples into different groups according to the median methDNA levels (Low: < 0.2; Partially: ≥ 0.2 and ≤ 0.6; High: > 0.6) of all promoter CG probesets (cg23464698 – cg14920289) and CG probesets within the body and 3’UTR regions (cg24736764 – cg08243827). The Kruskal-Wallis test and Dunn’s multiple comparisons test were applied to retrieve the statistical significance. FC and p-value are reported for each comparison group. *p-value*: * ≤ 0.05, **** < 0.0001

The gene/transcript expression and methDNA profiling of *SLC22A17* were also established by analyzing the TCGA Pan-Cancer SKCM datasets (Fig. 2A, B). The results indicated that the gene expression of ENSG00000092096.14, which included all the coding, non-coding, and retained intron transcripts, as well as the expression of ENST00000354772.7 and ENST00000206544.8 transcripts, were higher than 0 (median log2 = 3.073, 2.420, and 0.5859, respectively) (Fig. 2A). Moreover, the methDNA analysis showed that the CG probesets of the upstream promoter were hypomethylated (median Beta values < 0.2), whereas those within the downstream promoter were partially methylated (median Beta values = 0.21 for cg17199325 and 0.24 for cg14920289). Conversely, all the CG probesets belonging to the body and 3’UTR regions were globally hypermethylated (median Beta values ranged from 0.66 to 0.94) (Fig. 2B). Correlation analysis was also conducted to test if methDNA affects the *SLC22A17* expression in CM samples (Fig. 2C). Interestingly, all the CG probesets within the body and 3’UTR regions showed significant positive correlations (Pearson’s *R* ≥ 0.2, *p* ≤ 0.05) with the expression levels of ENSG00000092096.14, as well as all transcripts, except for ENST00000397267.5, ENST00000397260.7, and ENST00000474774.1. In addition, negative correlation pairs were observed between cg23464698, belonging to the upstream promoter region, and ENSG00000092096.14, ENST00000354772.7, ENST00000557699.5, and ENST00000206544.8 expression levels (Pearson’s *R* ≤ -0.2, *p* ≤ 0.05) (Fig. 2C).

To better understand the regulatory role of the CG probesets, taking into account their relative position within the *SLC22A17* locus, differential analysis of *SLC22A17* expression was performed by stratifying the TCGA Pan-Cancer SKCM samples into five groups according to the median methDNA levels of all the CG probesets pooled into promoter and body groups (Fig. 2D). Notably, no CM patients showed promoter hypermethylation and/or hypomethylation of the body region. The analysis revealed that *SLC22A17* expression was higher in CM samples belonging to the promoter-low/body-high group compared to the other groups, whereas the lowest expression levels were observed in the promoter-partially/body-partially group. Notably, the body-high groups displayed higher *SLC22A17* expression levels when compared to the body-partially groups. Among the body-high and body-partially groups, promoter hypomethylation was associated with the highest *SLC22A17* expression levels. These results indicated that the methDNA status of both the promoter and body regions significantly influenced the *SLC22A17* gene regulation (Fig. 2D).

### OS and PFI analyses of *SLC22A17* gene/transcript expression and methDNA in TCGA Pan-cancer SKCM cohort

**Fig. 3:**
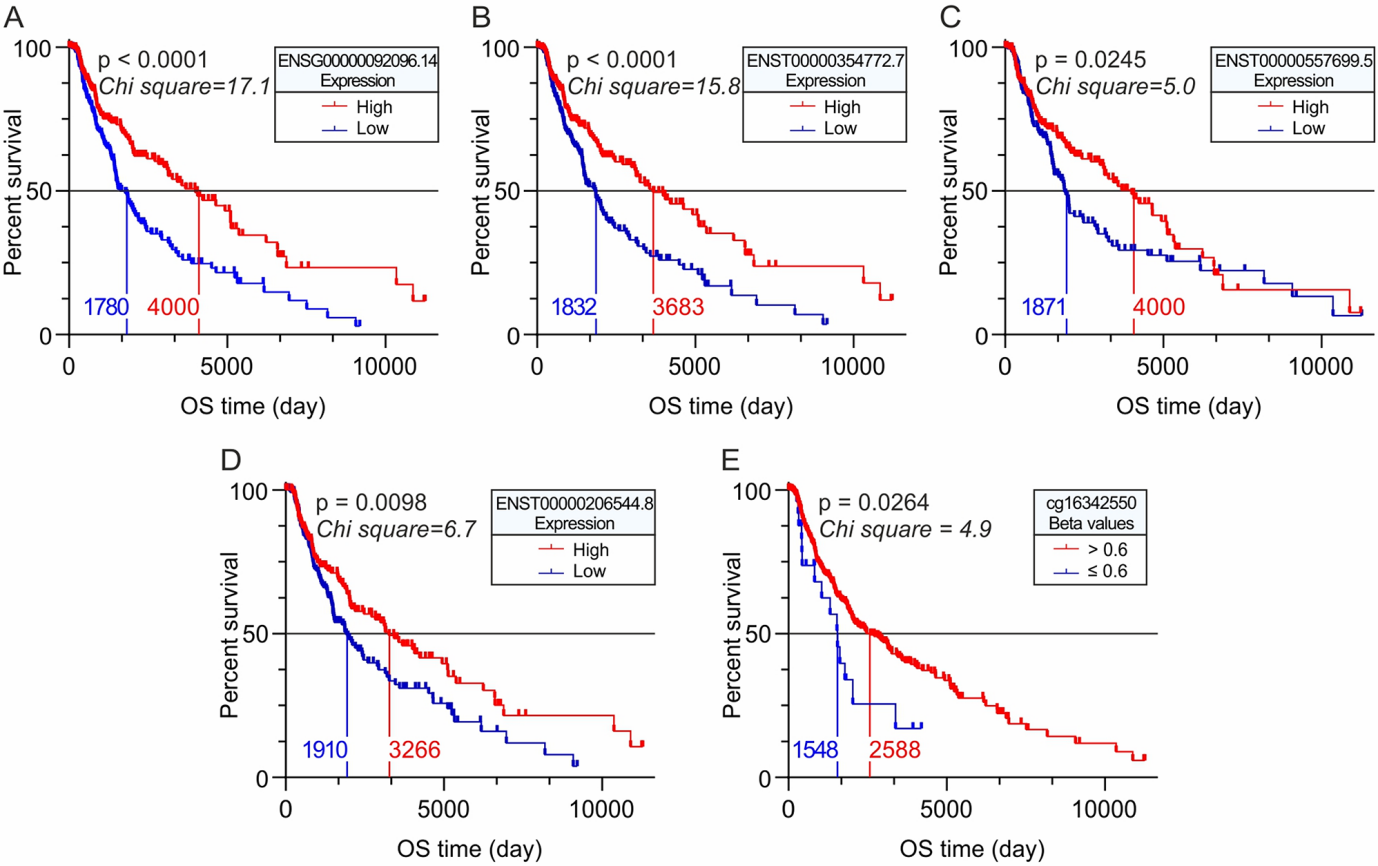
OS Kaplan-Meier analyses of *SLC22A17* expression and methDNA in the TCGA SKCM cohort. (**A-D**) The TCGA Pan-Cancer SKCM samples were stratified according to *SLC22A17* gene/transcript expression into high (≥ median value) and low (< median value) groups to estimate the difference in OS among these groups. (**E**) OS Kaplan-Meier analyses of SKCM samples stratified into hypermethylated (Beta value > 0.6) and partially/hypo-methylated groups (Beta value ≤ 0.6) according to methDNA levels of the cg16342550 probeset

**Fig. 4:**
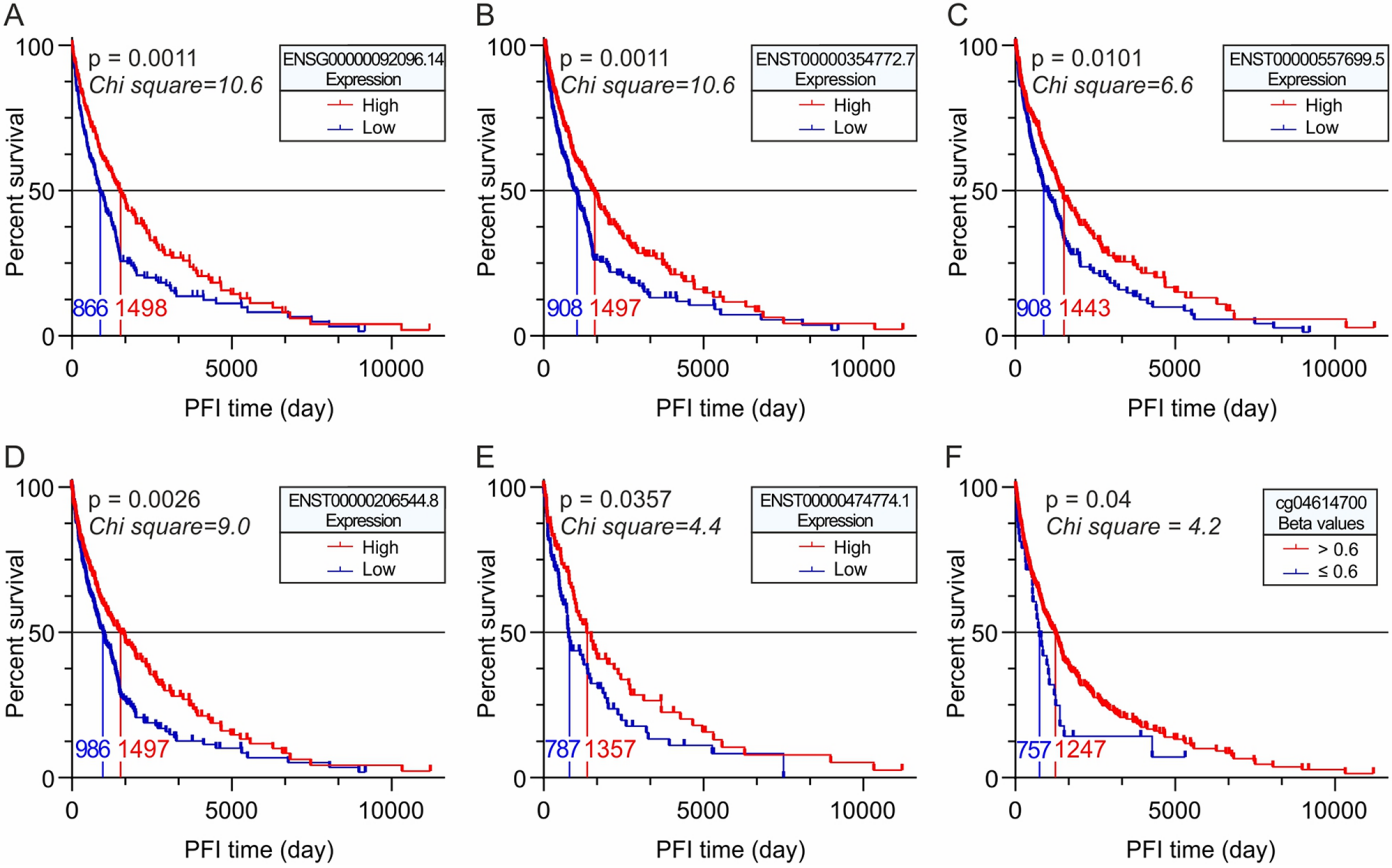
PFI Kaplan-Meier analyses of *SLC22A17* expression and methDNA in the TCGA SKCM cohort. (**A-E**) The TCGA Pan-Cancer SKCM samples were stratified according to *SLC22A17* gene/transcript expression into high (≥ median value) and low (< median value) groups to estimate the difference in PFI among these groups. (**F**) PFI Kaplan-Meier analysis was conducted on SKCM samples stratified into hypermethylated (Beta value > 0.6) and partially/hypo-methylated groups (Beta value ≤ 0.6) according to methDNA levels of the cg04614700 probeset

OS (Fig. 3) and PFI (Fig. 4) analyses of TCGA Pan-Cancer SKCM patients were performed using the Kaplan-Meier test, stratifying the samples according to *SLC22A17* gene/transcript expression in high (≥ median value) and low (< median value) groups, as well as in hypermethylated (Beta value > 0.6) and partially/low methylated (Beta value ≤ 0.6) groups according to the methDNA levels of *SLC22A17* CG probesets. As regards OS analyses, it was not possible to perform methDNA stratification for the cg01557297, cg19512521, and cg23411440 probesets due to the similar methDNA status of these CG probesets in all CM patients. OS and PFI Kaplan-Meier graphs of *SLC22A17* transcripts and CG probesets with no statistical significance were not reported.

The Kaplan-Meier analysis revealed that overexpression of the *SLC22A17* gene (ENSG00000092096.14) was associated with better OS and PFI for SKCM patients (Figs. 3A and 4A). Similarly, SKCM patients showing higher expression levels of ENST00000354772.7 (coding), ENST00000557699.5 (retained intron), and ENST00000206544.8 (coding) transcripts exhibited better prognosis than those with lower expression levels, considering both OS (Fig. 3B-D) and PFI (Fig. 4B-D). Regarding the non-coding ENST00000474774.1 transcript, its expression was only positively associated with PFI (Fig. 4E) in TCGA SKCM patients. Moreover, the Kaplan-Meier analyses performed on *SLC22A17* CG probesets showed that only the hypermethylation (Beta value > 0.6) of the body cg16342550 probeset was associated with better OS (Fig. 3E) in SKCM patients, while only the hypermethylation of the body cg04614700 probeset was positively associated with better PFI (Fig. 4F).

### Evaluation of *SLC22A17* expression and methDNA profiling in melanoma cell lines

**Fig. 5:**
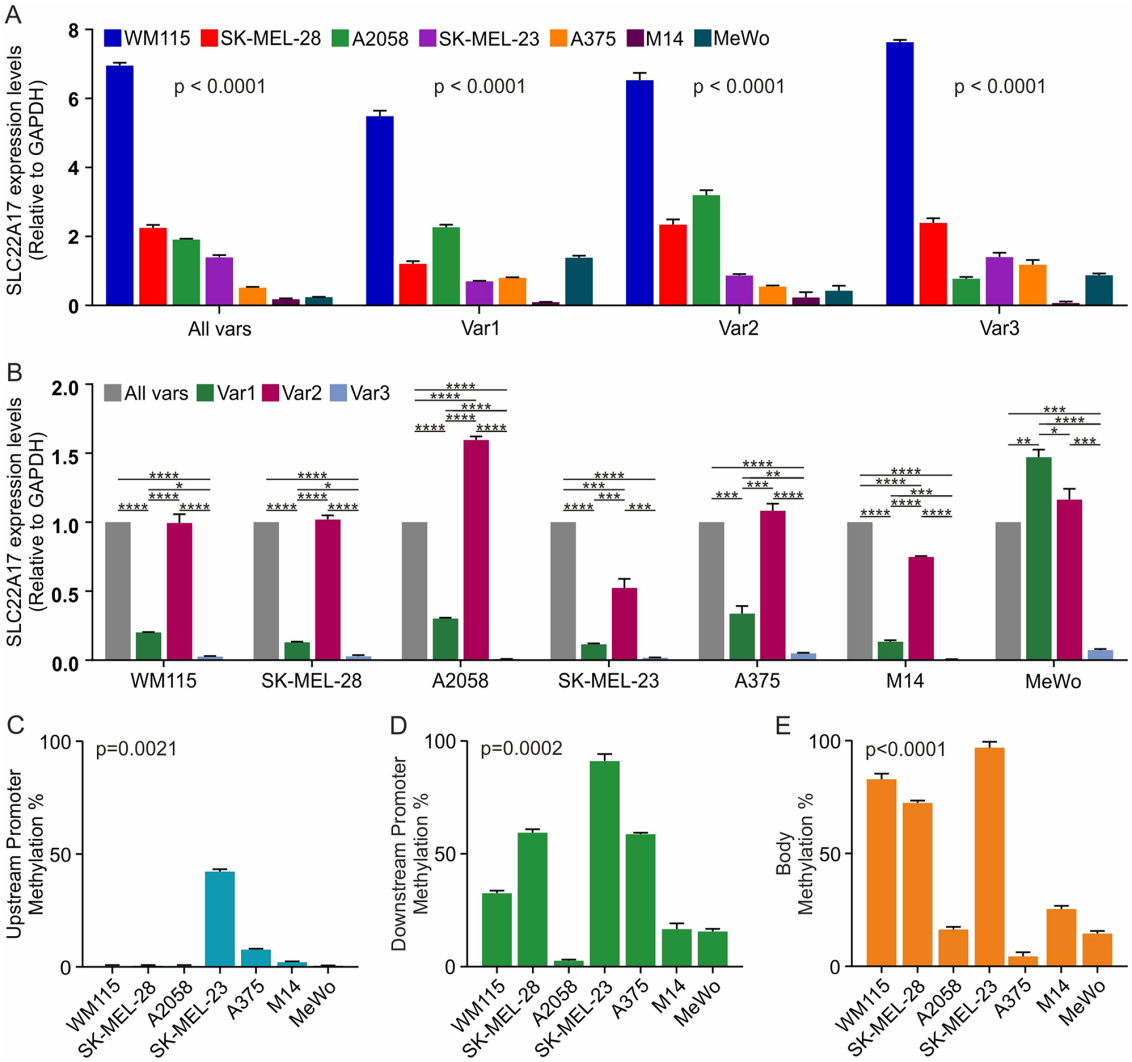
Gene/transcript expression and methDNA profiling of *SLC22A17* in melanoma cell lines. (**A**) The RT-qPCR analysis of *SLC22A17* expression was performed by amplifying Vars 1,2, and 3, as well as All Vars combined, in seven melanoma cell lines. The analysis of each target is reported comparing the expression levels of *SLC22A17* All Vars, as well as Vars 1, 2, and 3 among all melanoma cell lines. ΔΔCt was computed considering the mean values of all ΔCt (Ct *GAPDH* – Ct Target) for each target. Then, the FC was computed as $\:{2}^{-\varDelta\:\varDelta\:Ct}$ according to the ΔΔCt relative quantification method. The differential analysis was performed between *SLC22A17* Vars using the one-way ANOVA test. (**B**) The RT-qPCR expression analysis was also conducted by comparing the expression levels of Vars 1, 2, and 3 to All Vars combined within each melanoma cell line. The One-way ANOVA multiple comparisons test was used to evaluate the statistical significance (*p-value*: * ≤ 0.05, ** < 0.01, *** < 0.001, **** < 0.0001). (**C-E**) The MSRE-qPCR analysis was performed to evaluate the methDNA levels of three methDNA hotspots mapped in the upstream promoter, downstream promoter, and body regions. The Kruskal-Wallis test was performed for the comparison of methDNA levels of each hotspot among the melanoma cell lines

To better clarify the role of methDNA in *SLC22A17* gene regulation, both expression levels and methDNA status of *SLC22A17* were assessed in A375, A2058, SK-MEL-28, WM115, MeWo, M14, and SK-MEL-23 CM cells lines (Fig. 5). The *SLC22A17* expression analysis was carried out using two different approaches: (i) comparing the expression levels of All Var, Var 1, 2, and 3 among melanoma cell lines (Fig. 5A); (ii) comparing the expression levels of *SLC22A17* variants within each cell line (Fig. 5B). The RT-qPCR expression analysis of *SLC22A17* revealed that WM115 cells showed higher expression levels of *SLC22A17* All Vars, as well as Vars 1, 2, and 3 (FC = All Vars: 7.0; Var 1: 5.5; Var 2: 6.5; Var 3: 7.6) compared to other melanoma cell lines. The A2058 and SK-MEL-28 cells showed intermediate expression levels for all *SLC22A17* variants, while low expression levels were detected in the other cell lines (FC < 2) (Fig. 5A). Interestingly, Var 2 was the most expressed *SLC22A17* variant in all melanoma cell lines, except for MeWo, in which Var 1 showed the highest expression levels. Conversely, *SLC22A17* Var 3 was significantly downregulated in all the analyzed CM cell lines (Fig. 5B).

The methDNA status of the selected *SLC22A17* hotspots was determined using the MSRE-qPCR method, based on the HpaII/MspI digestion of DNA targets. The analysis showed that the *SLC22A17* upstream promoter hotspot was partially methylated only in SK-MEL-23 cells (42.3%), while low or undetectable methDNA levels were observed in other CM cell lines (≤ 7.9%) (Fig. 5C). Notably, the *SLC22A17* downstream promoter hotspot was hypermethylated in SK-MEL-23 (91%), partially methylated in A375, WM115, and SK-MEL-28 (58.8%, 32.6%, and 59.5%, respectively), and hypomethylated in A2058, MeWo, and M14 cells (≤ 16.8%) (Fig. 5D). Similarly, high methDNA levels of the *SLC22A17* body hotspot were detected in SK-MEL-23, WM115, and SK-MEL-28 (96.9%, 83%, and 72.5%, respectively), whereas low methDNA levels were observed in the other cell lines (≤ 25.8%) (Fig. 5E).

**Fig. 6:**
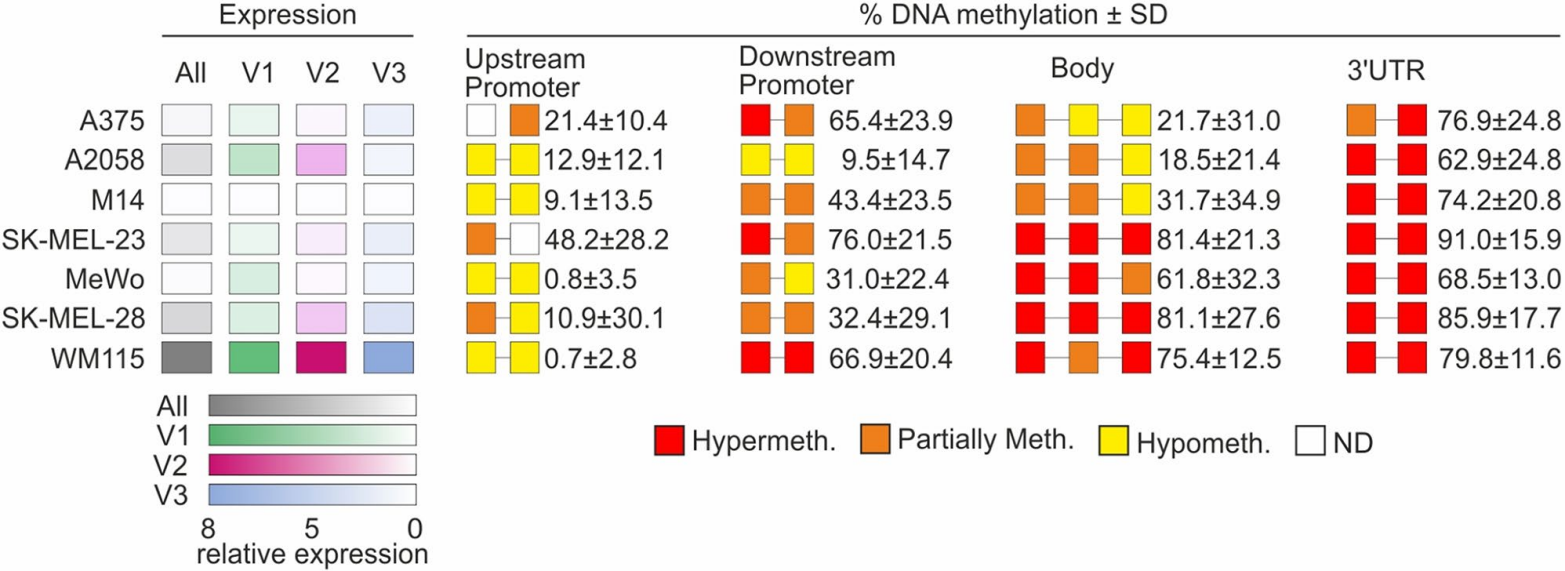
Heatmap representation of the relationship between *SLC22A17* expression and methDNA in melanoma cell lines. The left panel shows the expression levels of *SLC22A17* All Vars and Vars 1, 2, and 3 tested by RT-qPCR. The expression levels in all melanoma cell lines were normalized by the mean of ΔCt computed for each target. The right panel represents the mean ± SD values of methDNA levels of the CpG dinucleotides belonging to the promoter (upstream and downstream), body, and 3’UTR regions of *SLC22A17*. The methDNA analysis was performed by bisulfite conversion followed by PCR amplification and Sanger sequencing. Red, orange, and yellow boxes indicate hypermethylated (median Beta value > 0.6), partially methylated (0.2 ≤ median Beta value ≤ 0.6), and hypomethylated (median Beta value < 0.2) CpG groups, respectively. ND: Not detected

Bisulfite sequencing of nine sequences belonging to the promoter, body, and 3’UTR regions was also performed to analyze *SLC22A17* methDNA at single CpG resolution (Supplementary Fig. S2, S3). The mean and standard deviation (SD) of methDNA levels of 70 CpGs, clustered into nine CpGs groups (two groups for Up- and Downstream promoter, three for the body region, and two for the 3’UTR), were calculated in all melanoma cell lines included in this analysis. Furthermore, to evaluate the relationship between the methDNA profile and *SLC22A17* expression levels, the relative expression levels of All Vars analyzed together and individually (Vars 1, 2, and 3) were reported (Fig. 6).

The results indicated that the higher expression levels of *SLC22A17* detected in WM115 were associated with upstream promoter hypomethylation (0.7% ± 2.8), as well as downstream promoter and body hypermethylation (66.9% ± 20.4 and 75.4% ± 12.5, respectively). Similarly, hypermethylation of the body region was observed for SK-MEL-23 and SK-MEL-28 (81.4% ± 21.3 and 81.1% ± 27.6, respectively), while the *SLC22A17* expression levels were lower in these cell lines compared to WM115 cells, probably due to the partially methylated status of the upstream promoter in SK-MEL-23 (48.2% ± 28.2) and downstream promoter in SK-MEL-28 (32.4% ± 29.1) (Fig. 6). Among the other melanoma cell lines, A2058 showed the highest *SLC22A17* expression levels, followed by A375 cells. In particular, the methDNA profile of A2058 cell line was characterized by low methDNA status of the upstream and downstream promoter regions (12.9% ± 12.1 and 9.5% ± 14.7, respectively) and medium/low methDNA levels of the body region (18.5% ± 21.4), while A375 showed medium/low methDNA levels of both upstream/downstream promoter and body regions (21.4% ± 10.4, 65.4% ± 23.9, and 21.7% ± 31.0, respectively) (Fig. 6). Finally, the lowest *SLC22A17* expression levels detected in the M14 and MeWo cells were associated with hypomethylation of the upstream promoter (9.1% ± 13.5 for M14 and 0.8% ± 3.5 for MeWo) and medium/low methDNA levels of the downstream promoter (43.4% ± 23.5 for M14 and 31.0% ± 22.4 for MeWo) and body regions (31.7% ± 34.9 for M14 and 61.8% ± 32.3 for MeWo). Since the 3’UTR region was hypermethylated (≥ 62.9%) in all melanoma cell lines, no relevant regulatory role in *SLC22A17* gene expression was observed, indicating that the *SLC22A17* expression levels mainly depend on the methDNA status of the promoter and body regions (Fig. 6). The correlation analysis between *SLC22A17* expression (All Vars, Var 1, Var 2, and Var 3) and the mean methDNA levels of all the CG probesets grouped by region (Upstream promoter, Downstream promoter, Body, and 3’ UTR) was also performed; however, no statistical significance was achieved (data not shown).

### *SLC22A17* expression and methDNA in 5-Aza-treated melanoma cell lines

**Fig. 7:**
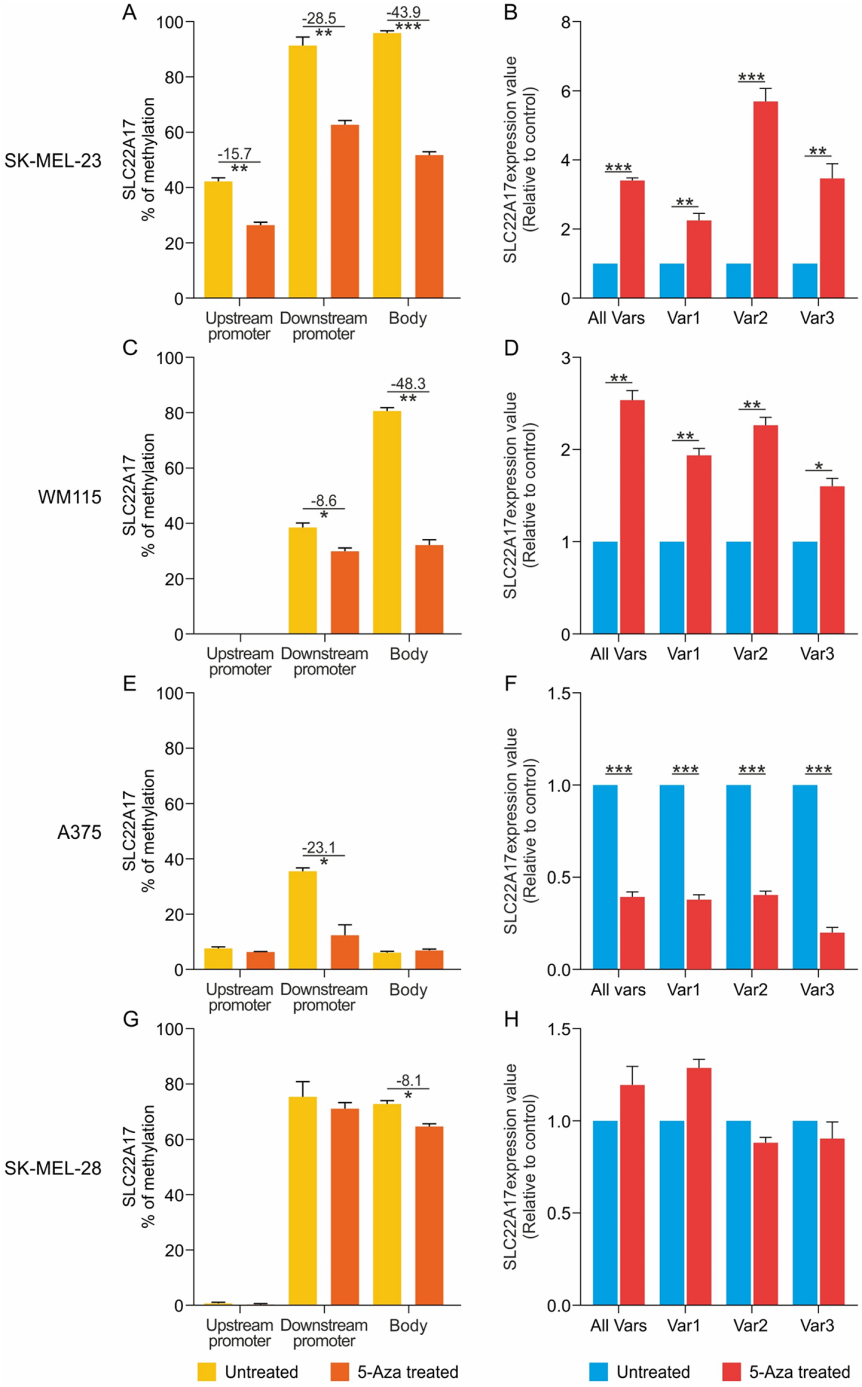
Expression and methDNA analyses of *SLC22A17* in 5-Aza-treated melanoma cell lines. (**A**,** C**,** E**, and **G**) MethDNA analysis of upstream, downstream promoter, and body hotspots in 5-Aza treated and untreated melanoma cell lines. The difference in the methDNA levels between treated and untreated cells is reported as Beta difference. The differential analysis was performed using the Mann-Whitney test. (**B**,** D**,** F**, and **H**) *SLC22A17* expression analysis of All Vars and Vars 1, 2, and 3 in 5-Aza treated cells compared to controls. The statistical significance was assessed using a paired two-tailed T-test. *p-value*: * ≤ 0.05, ** < 0.01, *** < 0.001

Functional experiments were performed to modulate the methDNA status in four melanoma cell lines and demonstrate the correlation between the expression and methDNA of *SLC22A17*. In particular, the SK-MEL-23, WM115, A375, and SK-MEL-28 cell lines were treated with the demethylating agent 5-Aza at concentrations of 1.9 µM for SK-MEL-23, 0.75 µM for WM115 and A375, and 3.5 µM for SK-MEL-28, while the M14, A2058, and MeWo cells were excluded from 5-Aza treatment due to the low methDNA status of the upstream/downstream promoter and body hotspots (Fig. 7 and Supplementary Fig. S1).

The results indicated that the most significant methDNA reduction of upstream and downstream promoter and body hotspots was obtained in the 5-Aza-treated SK-MEL-23 cell line (% of methDNA reduction: 15.7%, 28.5%, and 43.9%, respectively; *p* < 0.01). In this cell line, strong upregulation of *SLC22A17* All Vars was observed, along with the upregulation of the three variants analyzed independently (Vars 1, 2, and 3) (FC: from 2.3 to 5.7, *p* < 0.01) (Fig. 7A, B). Similarly, 5-Aza treatment induced DNA demethylation of the downstream promoter and body methDNA hotspots in WM115 (downstream promoter: 8.6%, *p* ≤ 0.05; body: 48.3%, *p* < 0.01), as well as significant upregulation of *SLC22A17* All Vars/Vars 1, 2, and 3 in treated compared to untreated cells (FC: from 1.6 to 2.5, *p* ≤ 0.05). Notably, the upstream promoter hotspot was unmethylated in both 5-Aza-treated and untreated WM115 (Fig. 7C, D). An opposite trend was observed for A375 cells, in which *SLC22A17* expression levels were significantly downregulated (> 50%, *p* < 0.001) in treated compared to control cells. Since methDNA levels were low (< 10%) in the upstream promoter and body methDNA hotspots and moderate in the downstream promoter, the downregulation of *SLC22A17* observed in A375 may be due to the negative modulation of the *SLC22A17* pathway, independently of the demethylation of *SLC22A17* hotspots (Fig. 7E, F). Finally, no significant variation was detected neither for methDNA hotspots nor for the *SLC22A17* All Vars/Vars 1, 2, and 3 expression in SK-MEL-28 cells, which were more resistant to 5-Aza treatment compared to the other melanoma cell lines (Fig. 7G, H and Supplementary Fig. S1).

### MSRE-ddPCR analysis of the *SLC22A17* downstream promoter hotspot in FFPE CM and nevi samples

**Fig. 8:**
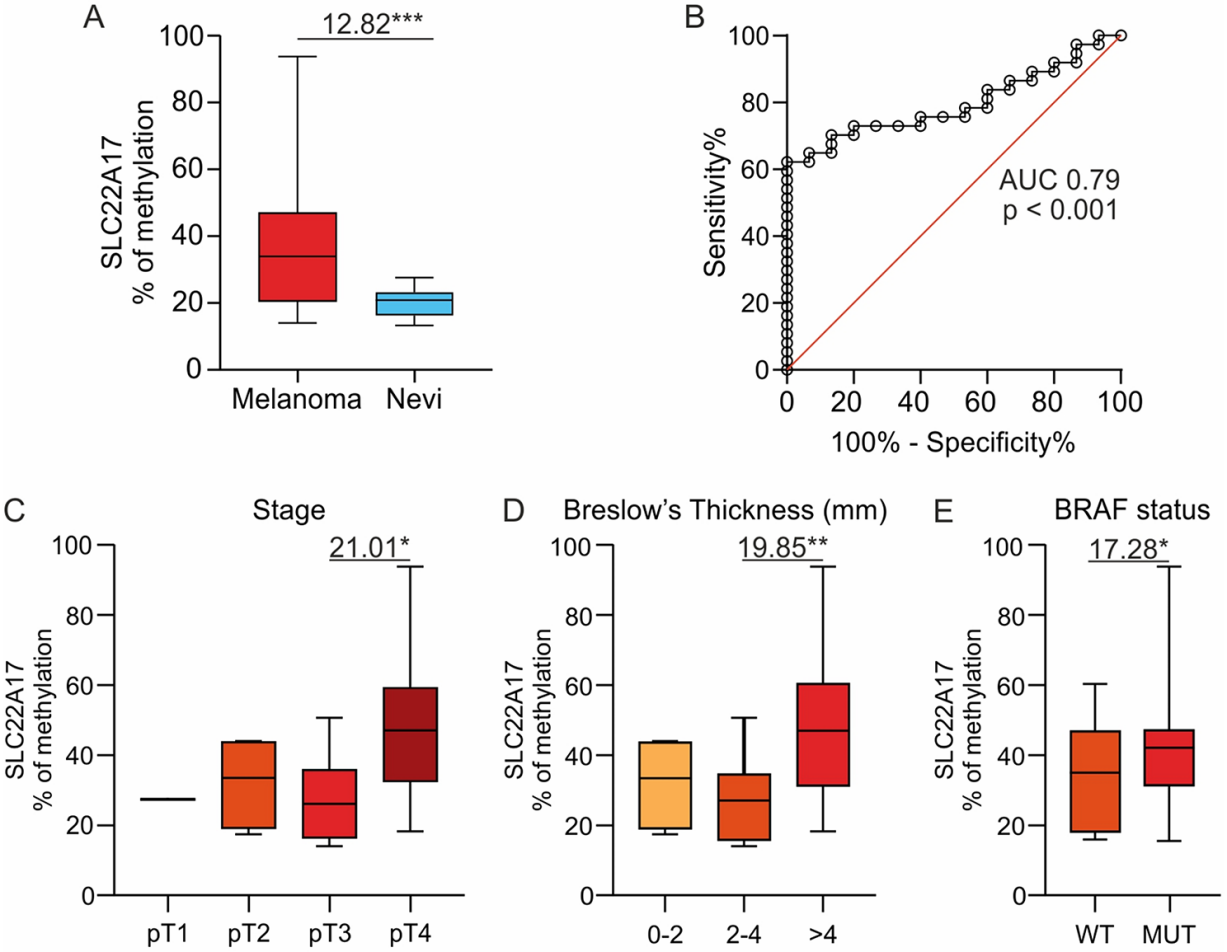
MethDNA analysis of the *SLC22A17* downstream promoter hotspot in CM and nevi FFPE samples. (**A**) MethDNA levels of the *SLC22A17* downstream promoter hotspot were analyzed in CM tissue samples (*N* = 37) and benign nevi (*N* = 15) using the custom MSRE-ddPCR assay. The differential analysis was performed using the Mann-Whitney test. (**B**) Diagnostic test performance was performed by ROC analysis. AUC and *p-value* are indicated. (**C-E**) The differential analysis of the *SLC22A17* downstream promoter hotspot was performed by stratifying CM samples according to stage, Breslow Thickness (mm), and BRAF status. The Mann-Whitney test was used for comparing two groups, whereas the Kruskal-Wallis test and Dunn’s multiple comparisons test were performed for the analyses of more than two groups. *p-value*: * ≤ 0.05, ** < 0.01 *** < 0.001

To evaluate the translational relevance of the in silico and in vitro results, methDNA levels of the selected *SLC22A17* hotspot, belonging to the downstream promoter region, were analyzed in 37 CM and 15 nevi samples (Fig. 8). To this end, the custom MSRE-ddPCR assay was performed on FFPE specimens, demonstrating that methDNA levels of the *SLC22A17* hotspot were significantly higher in CM compared to nevi samples (median methDNA percentage = 33.72% vs. 20.90%; *p* < 0.001) (Fig. 8A). Moreover, ROC analysis revealed that the *SLC22A17* methDNA hotspot had good biomarker performance (AUC = 0.79, *p* < 0.001, cut off = 27.33%), suggesting its potential application as a diagnostic biomarker for CM (Fig. 8B).

The methDNA levels of the *SLC22A17* hotspot were also evaluated by stratifying FFPE CM and nevi samples according to the available socio-demographic characteristics (age and gender); however, no significant variation was observed among the considered groups (Supplementary Fig. S4A, S4B). In addition, CM samples were stratified according to the main clinical-pathological features, including stage, Breslow thickness, BRAF status, number of mitosis, Tumor-Infiltrating Lymphocytes (TILs), vascular invasion, and ulceration (Fig. 8C-E and Supplementary Fig. S4C-F). The analysis revealed that methDNA levels of the *SLC22A17* downstream promoter hotspot were higher in the pT4 group (median: 47.0%, Q1-Q3: 32.4–59.1) compared to the pT1-pT3 groups (pT1: median: 27%; pT2: median: 33.4%, Q1-Q3: 19.1–43.6; pT3: median: 26.0% Q1-Q3:15.8–35.8, respectively), showing a statistically significant difference (median methDNA difference = 21.01%; *p* ≤ 0.05) only for the pT3 and pT4 comparison groups (Fig. 8C). Similarly, the CM samples with Breslow’s thickness > 4 showed higher methDNA levels compared to those with thickness < 2 (median methDNA difference: 12.6%, ns) and thickness 2–4 (median methDNA difference: 19.85%, *p* < 0.01) groups (Fig. 8D). Interestingly, a significant increment in methDNA of the *SLC22A17* downstream promoter hotspot was observed in BRAF mutated compared to BRAF wild type (WT) CM specimens (median methDNA difference: 17.28%, *p* ≤ 0.05) (Fig. 8E). Regarding the other clinical-pathological features, only vascular invasion and ulceration status showed a trend, not statistically significant, in which the methDNA levels of the *SLC22A17* hotspot were increased in advanced CM patients (Supplementary Fig. S4E, S4F).

## Discussion

It is known that both genetic and environmental factors are associated with an increased risk of CM [35–38]. Besides genetic mutations, epigenetic regulatory mechanisms are involved in CM initiation and progression [39, 40]. Among epigenetic alterations, methDNA plays a pivotal role in the regulation of cancer-related genes. The relationship between methDNA and CM development and immune response has been previously investigated, highlighting the diagnostic and prognostic potential of aberrant methDNA [41, 42]. For instance, Gao and colleagues showed that the promoter hypermethylation of *Claudin 11* (*CLDN11*) can be used to discriminate between CM and dysplastic nevi, thus representing a potential melanoma-specific epigenetic biomarker [43]. Similarly, Rius et al. investigated the methDNA landscape of invasive melanoma cells and identified a signature of genes with promoter hypermethylation and decreased expression, including *Rho-Type GTPase-Activating Protein 22* (*ARHGAP22*) and *Neuron Navigator 2* (*NAV2*), which were correlated with poor survival in CM patients [44]. Integrative analyses of the methylome also revealed that gene body methDNA status was associated with distinct phenotypic features that predicted the OS of CM patients [45, 46].

In the last few years, many studies have demonstrated that aberrant methDNA influences the TME, promoting tumor growth and progression [47–52]. In this context, our previous research showed the role of methDNA in the modulation of the *NGAL/SLC22A17/MMP-9* network in several tumor types [31]. Over the years, it was widely demonstrated that the activation of the *SLC22A17* gene partners (*NGAL* and *MMP-9*) may promote different malignancies, including glioma, endometrial cancer, lung adenocarcinoma, and gastric cancer [53–56]. Moreover, recent studies highlighted that the aberrant gene expression of *NGAL* and *MMP-9* is strictly regulated by methDNA status in several pathological conditions, including cancer [57–60]. Similarly, *SLC22A17* overexpression plays a critical role in the pathogenesis of gastric and non-small cell lung cancer, representing a negative prognostic biomarker for such tumors [30, 61, 62]. Previous studies demonstrated that the aberrant expression of *SLC22A17* is strictly associated with poor clinical outcomes in patients with endometrial carcinoma, glioma, and hepatocellular carcinoma (HCC) [63–65]. Interestingly, Gomez-Chou and colleagues also reported that the downregulation of *SLC22A17* significantly reduced the mRNA expression levels of the pro-inflammatory cytokines and MMPs in pancreatic cancer cells [66]. In contrast, *SLC22A17* overexpression is associated with a reduced risk of urinary tract infections and renal cancer [29, 67].

The different prognostic significance of *SLC22A17* overexpression in various tumor types leads us to analyze the mRNA expression levels of *SLC22A17* in CM, where its clinical implications are still unknown. On these bases, in the present study, the relationship between methDNA and *SLC22A17* expression in CM was investigated through computational approaches and further studied in both melanoma cell lines and tissue samples derived from CM patients.

The computational analysis showed that the mRNA expression levels of *SLC22A17* were significantly lower in CM samples compared to those of nevi samples, suggesting that *SLC22A17* may act as a tumor suppressor gene. The obtained results highlighted the crucial role of both the downstream promoter and body regions methDNA status in the regulation of *SLC22A17* expression. In particular, higher methDNA levels in downstream promoter and lower methDNA status in the body region were associated with *SLC22A17* downregulation in CM compared to nevi, However, the CG probesets in the upstream promoter region appeared not to have a regulatory role in this context. These results are consistent with those previously reported, in which *SLC22A17* downregulation is strictly related to the initiation, progression, and drug resistance of different tumor types [29, 31, 32].

Interestingly, we observed that the expression pattern of *SLC22A17* appeared to be strongly associated with the methDNA status of both the promoter and body regions. In particular, the expression analysis of the TCGA SKCM dataset showed that CM samples with promoter hypomethylation and body hypermethylation exhibited the highest *SLC22A17* expression levels compared to other groups. These findings are in agreement with previous observations in which *SLC22A17* expression is strictly regulated by methDNA status in childhood acute lymphoblastic leukemia and chronic musculoskeletal pain [68, 69]. OS and PFI analyses of the same dataset revealed that both the overexpression of *SLC22A17* and the hypermethylation of the body region significantly enhanced the survival rate of CM patients.

The biological significance of *SLC22A17* overexpression observed through computational approaches was investigated by in vitro studies, revealing that *SLC22A17* overexpression is strictly associated with intragenic hypermethylation and promoter region hypomethylation. CM cells treated with the demethylating agent (5-Aza) showed that promoter demethylation significantly increased *SLC22A17* expression, especially in the SK-MEL-23 and WM115 cell lines. Despite the reduction of body methDNA levels observed in 5-Aza-treated cells, SK-MEL-23 and WM115 maintained a partial methDNA status in the body region, which sustained *SLC22A17* overexpression along with promoter demethylation. Similar data were obtained in the HCC model, in which the authors performed pharmacological unmasking of epigenetically silenced tumor suppressor genes [70]. Among the analyzed genes, *SLC22A17* was significantly upregulated in HCC cell lines after treatment with the demethylating agent 5-aza-2’-deoxycytidine (5-Aza-dC) and the histone deacetylase inhibitor trichostatin A (TSA), corroborating the evidence that promoter hypomethylation is crucial for the activation of *SLC22A17* [70].

To better understand the clinical significance of the in silico and in vitro results, methDNA levels of the *SLC22A17* downstream promoter hotspot were analyzed in FFPE samples obtained from a group of CM patients and healthy controls. The analysis revealed that methDNA levels of the *SLC22A17* hotspot allowed for discrimination between CM patients and healthy individuals. Accordingly, hypermethylation of the *SLC22A17* hotspot was also associated with advanced CM, especially when considering stage and Breslow’s thickness. To the best of our knowledge, the reported findings demonstrate for the first time that hypermethylation of the *SLC22A17* promoter hotspot may represent not only a promising epigenetic hallmark of cancer initiation but also a putative methDNA-based biomarker to predict worse prognosis for CM patients. Overall, the results here obtained represent a starting point for the validation of other *SLC22A17* CG probesets as a diagnostic and prognostic panel in CM. Similar analyses could be also extended to *SLC22A17*-related genes to strengthen its potential application in clinical practice. Moreover, further validation studies in a larger cohort of CM patients and healthy controls are needed to corroborate our preliminary findings, also in other biological samples (e.g. metastasis or liquid biopsy samples), considering the follow-up and treatment response data that are missing in the CM samples analyzed in the present study.

## Conclusions

Overall, the present study demonstrates that the methDNA status of both promoter and intragenic regions significantly affects *SLC22A17* expression levels in CM. Notably, promoter hypermethylation is associated with the transcriptional silencing of *SLC22A17*, while methDNA levels of body CG probesets are positively related to its expression. MethDNA levels of the investigated *SLC22A17* hotspot, belonging to the downstream promoter region, increase during both the initiation and progression of CM, indicating its potential role as an epigenetic biomarker in CM management. These preliminary results pave the way for the identification of additional methDNA hotspots with diagnostic and prognostic value for CM and other malignancies.

## Electronic supplementary material

Below is the link to the electronic supplementary material.

Supplementary Material 1: Evaluation of melanoma cell line sensitivity to 5-Aza treatment. WM115, SK-MEL-23, SK-MEL-28 (seed density of 4 × 10^3^ cells per 96-well), and A375 (2 × 10^3^ per 96-well), were treated with serial dilutions of 5-Aza (100 − 10–1 − 0.1–0.01 − 0.001 µM) for 72 h. Cell viability was evaluated by MTT assay. GraphPad Prism (version 8.0.2) was used to calculate the mean and SD of 5-Aza IC_50_ concentrations for each melanoma cell line

Supplementary Material 2: UCSC visualization of the *SLC22A17* locus. The *SLC22A17* CG probesets (Infinium 450 K Bead array) included in the bioinformatic analysis are displayed. The sequences used for bisulfite-Sanger sequencing and PCR-based MSRE analyses were aligned to the *SLC22A17* genomic sequence

Supplementary Material 3: Bisulfite conversion and Sanger sequencing analysis of *SLC22A17* in melanoma cell lines. (**A-D**) MethDNA analysis of CpG hotspots included in the sequences Prom 1, 2, 3, and 4 within the *SLC22A17* promoter region. (**E**) MethDNA analysis of CpG hotspots belonging to the body region. (**F-G**) MethDNA levels of CpGs located in the 3’UTR region. Yellow circles indicate the CpG hotspots. Dark, gray, and yellow bars represent the sequenced fragments, the primers used for amplification, and CCGG restriction sites, respectively. The pie charts indicate the methDNA status for each CpG, reporting the percentage of methylation (red) and unmethylation (green). Gray pie charts refer to the undetected CpG methDNA levels. The methDNA percentage of each CG probeset was computed as the ratio between the height of the cytosine (unconverted CpG cytosine – methylated) and thymine (converted CpG cytosine – unmethylated) peaks retrieved from the sequence chromatogram obtained for each target

Supplementary Material 4: Differential analysis of the *SLC22A17* downstream promoter methDNA hotspot according to socio-demographic and clinical-pathological features. (**A-B**) The methDNA levels of the *SLC22A17* downstream promoter hotspot in CM and nevi tissues were analyzed by stratifying the FFPE samples according to age and gender. (**C-F**) CM tissues were also stratified according to the number of mitosis, Tumor-Infiltrating Lymphocytes (TILs), vascular invasion, and ulceration. The Mann-Whitney test was used for comparing two groups, whereas the Kruskal-Wallis test and Dunn’s multiple comparisons test were performed for the analyses of more than two groups

## Data Availability

Data from different centers will be shared differently according to different local regulatory requirements. Those deidentified data that are not readily shared will be made available upon reasonable request and provided in accordance with the corresponding regulatory requirements. Data from Fondazione “G. Pascale” of Naples and raw data of bisulfite sequencing analysis of *SLC22A17* DNA methylation hotspots in melanoma cell lines are available in a public, open-access repository at the following external link 10.5281/zenodo.13152503. Derived data supporting the findings of this study are available from the corresponding author [ML] upon reasonable request.

## Abbreviations

5-Aza: 5-Azacytidine
5-Aza-dC: 5-aza-2’-deoxycytidine
5mC: 5-methylcytosine
All vars: All variants
ARHGAP22: Rho-Type GTPase-Activating Protein 22
AUC: Area Under Curve
CH_3_: Methyl group
CLDN11: Claudin 11
CM: Cutaneous Melanoma
CpG: Cytosine-Guanine dinucleotide
ddPCR: Droplet digital PCR
DNTPs: Deoxynucleotide Triphosphates
ECM: Extracellular Matrix
FBS: Fetal Bovine Serum
FC: Fold Change
FFPE: Formalin-Fixed Paraffin-Embedded
GAPDH: Glyceraldehyde-3-Phosphate Dehydrogenase
GEO: Gene Expression Omnibus
LCN2: Lipocalin 2
methCTRL: methylation CTRL
methDNA: DNA methylation
MMPs: Metalloproteinases
MSRE: Methylation-sensitive restriction enzyme
N/A: Not Applicable
NAV2: Neuron Navigator 2
NGAL: Gelatinase-Associated Lipocalin
NGALR: NGAL receptor
NS: Not Significant
OD: Optical Density
OS: Overall Survival
PFI: Progression Free Interval
Q1: 1st Quartile
Q3: 3rd Quartile
ROC: Receiver Operating Characteristic
SAM: S-adenosyl-L-methionine
SD: Standard deviation
SKCM: Melanoma
SLC22A17: Solute Carrier Family 22 Member 17
SNPs: Single Nucleotide Polymorphisms
TCGA: The Cancer Genome Atlas
TILs: Tumor-Infiltrating Lymphocytes
TME: Tumor Microenvironment
TSA: Trichostatin A
Var 1: Variant 1
Var 2: Variant 2
Var 3: Variant 3
