# Supplementary figures and images for: Identification of SLC22A17 DNA methylation hotspot as a potential biomarker in cutaneous melanoma
Source: J Transl Med. 2024 Oct 2;22:887. doi: 10.1186/s12967-024-05622-9 (PMC11445995; doi:10.1186/s12967-024-05622-9)

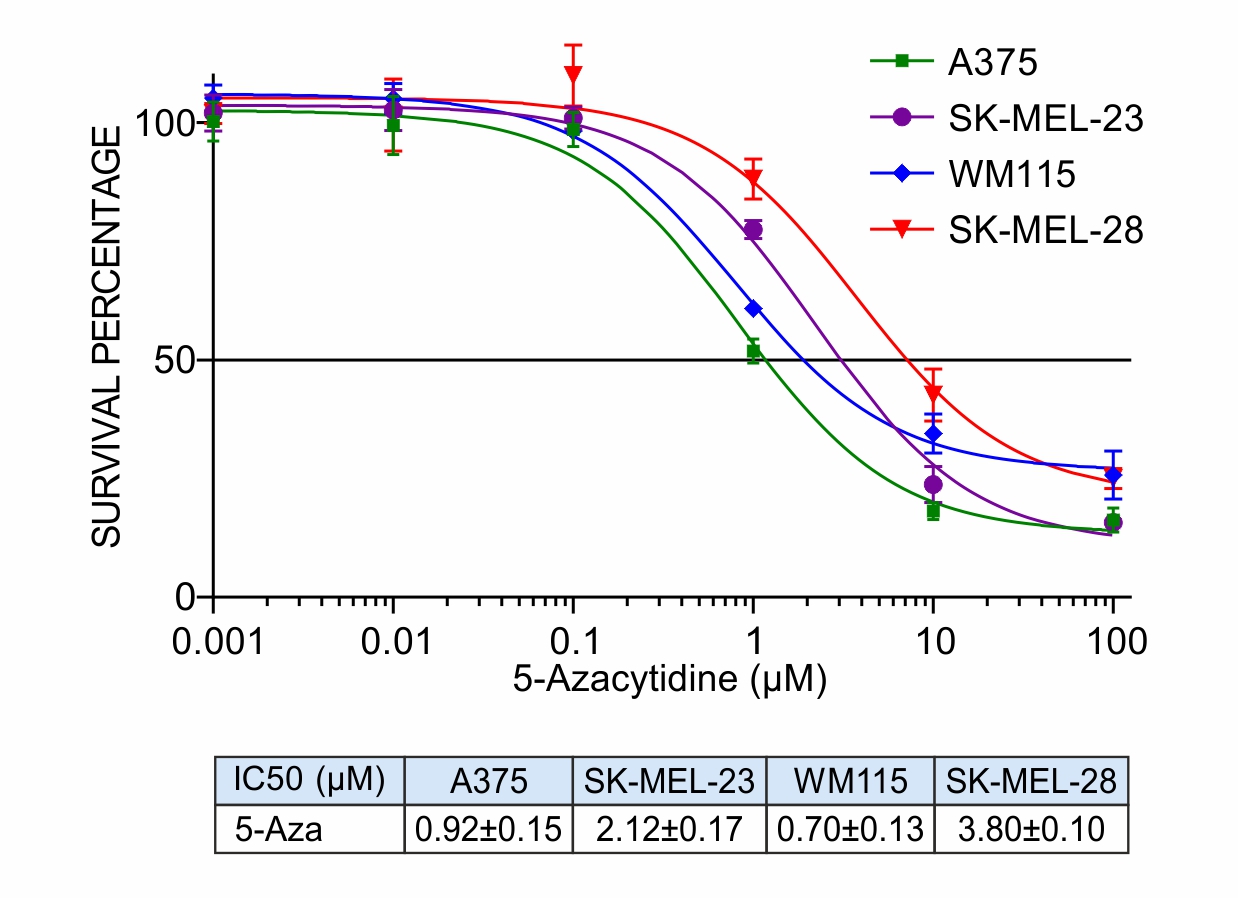

Supplement: Supplementary file 1 — Supplementary Material 1: Evaluation of melanoma cell line sensitivity to 5-Aza treatment. WM115, SK-MEL-23, SK-MEL-28 (seed density of 4 × 10^3^ cells per 96-well), and A375 (2 × 10^3^ per 96-well), were treated with serial dilutions of 5-Aza (100 − 10–1 − 0.1–0.01 − 0.001 µM) for 72 h. Cell viability was evaluated by MTT assay. GraphPad Prism (version 8.0.2) was used to calculate the mean and SD of 5-Aza IC50 concentrations for each melanoma cell line [file 12967_2024_5622_MOESM1_ESM.jpg]

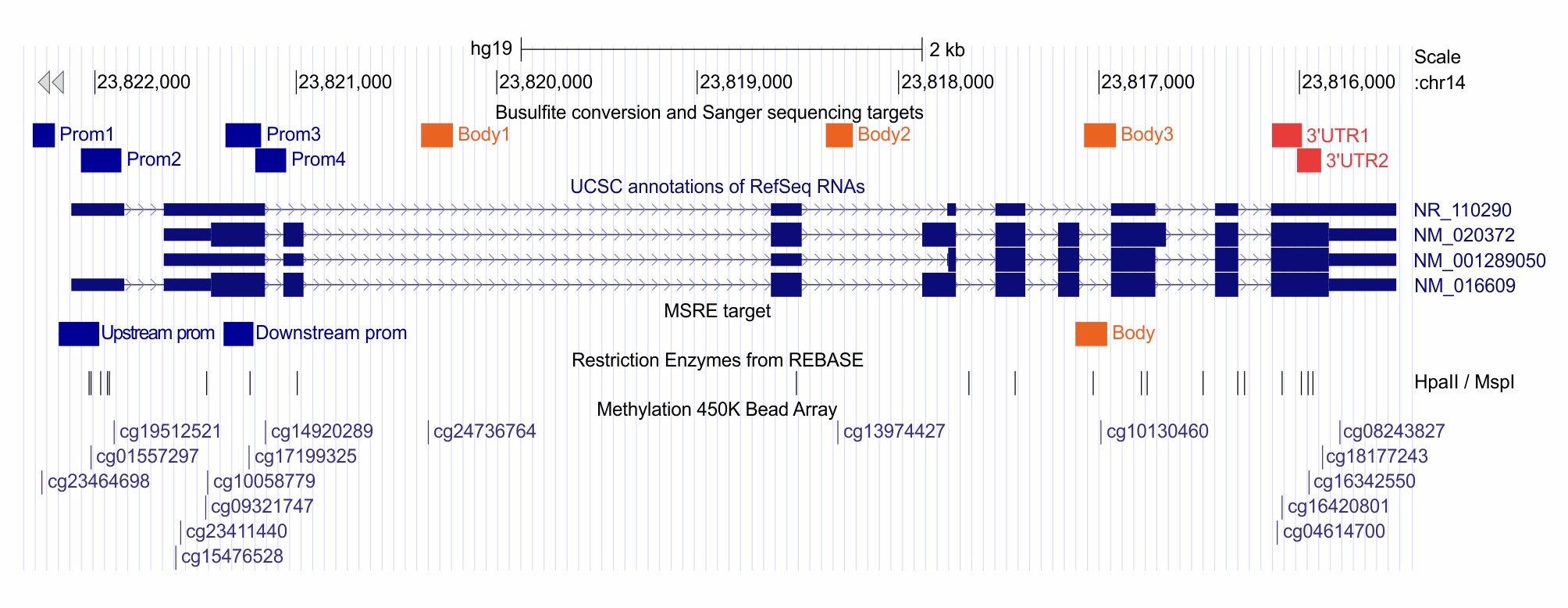

Supplement: Supplementary file 2 — Supplementary Material 2: UCSC visualization of the SLC22A17 locus. The SLC22A17 CG probesets (Infinium 450 K Bead array) included in the bioinformatic analysis are displayed. The sequences used for bisulfite-Sanger sequencing and PCR-based MSRE analyses were aligned to the SLC22A17 genomic sequence [file 12967_2024_5622_MOESM2_ESM.jpg]

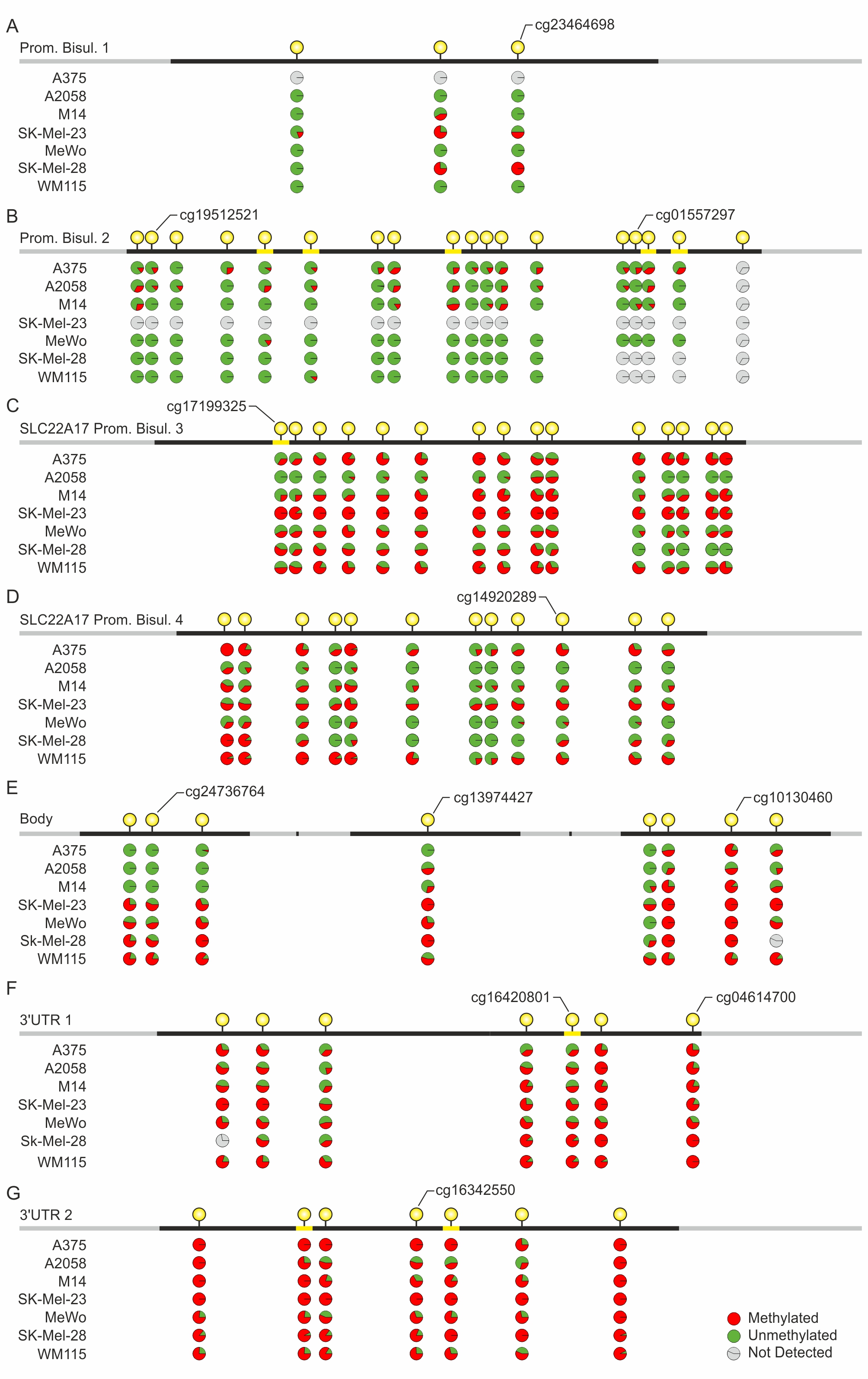

Supplement: Supplementary file 3 — Supplementary Material 3: Bisulfite conversion and Sanger sequencing analysis of SLC22A17 in melanoma cell lines. (A-D) MethDNA analysis of CpG hotspots included in the sequences Prom 1, 2, 3, and 4 within the SLC22A17 promoter region. (E) MethDNA analysis of CpG hotspots belonging to the body region. (F-G) MethDNA levels of CpGs located in the 3’UTR region. Yellow circles indicate the CpG hotspots. Dark, gray, and yellow bars represent the sequenced fragments, the primers used for amplification, and CCGG restriction sites, respectively. The pie charts indicate the methDNA status for each CpG, reporting the percentage of methylation (red) and unmethylation (green). Gray pie charts refer to the undetected CpG methDNA levels. The methDNA percentage of each CG probeset was computed as the ratio between the height of the cytosine (unconverted CpG cytosine – methylated) and thymine (converted CpG cytosine – unmethylated) peaks retrieved from the sequence chromatogram obtained for each target [file 12967_2024_5622_MOESM3_ESM.jpg]

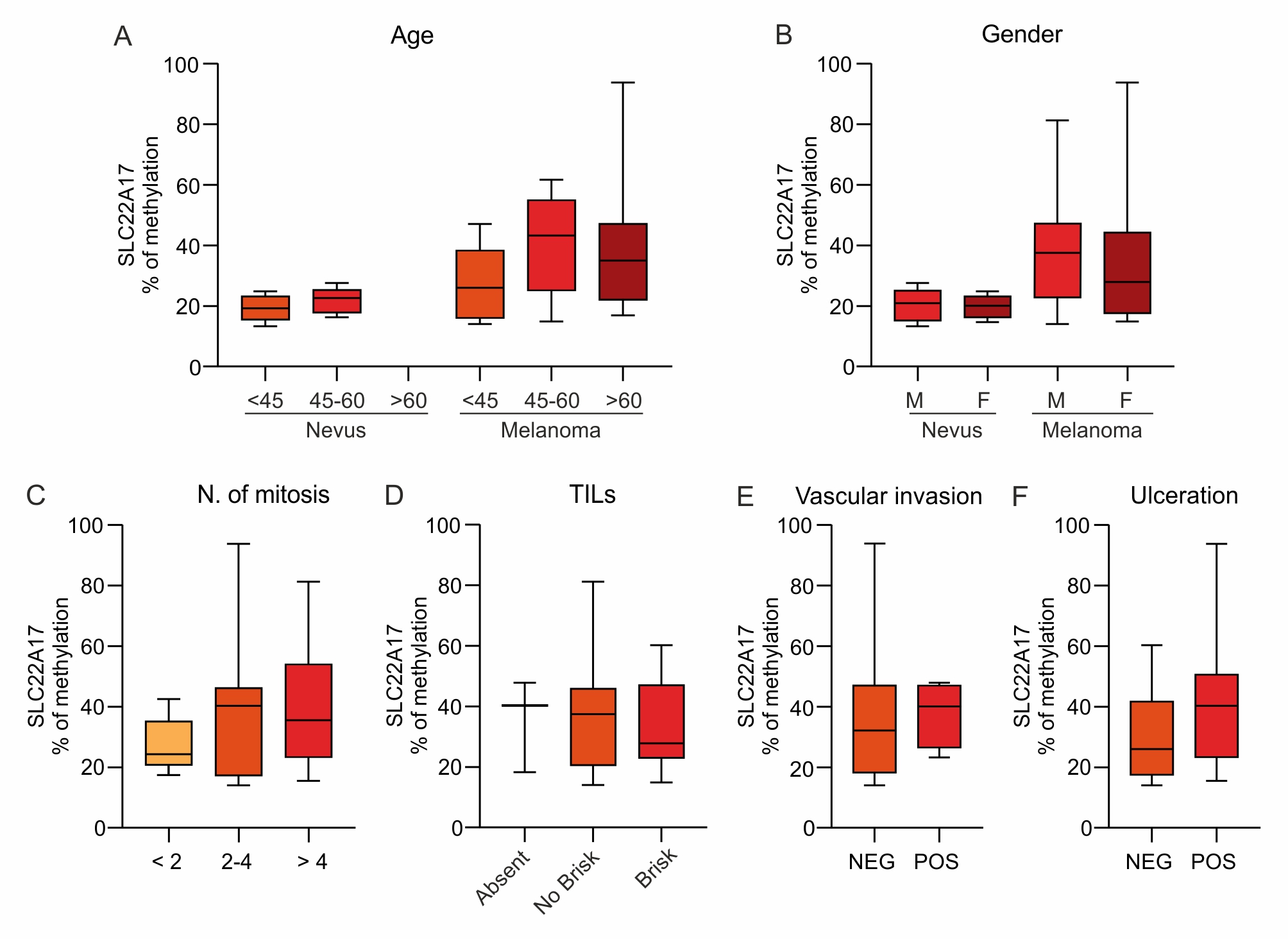

Supplement: Supplementary file 4 — Supplementary Material 4: Differential analysis of the SLC22A17 downstream promoter methDNA hotspot according to socio-demographic and clinical-pathological features. (A-B) The methDNA levels of the SLC22A17 downstream promoter hotspot in CM and nevi tissues were analyzed by stratifying the FFPE samples according to age and gender. (C-F) CM tissues were also stratified according to the number of mitosis, Tumor-Infiltrating Lymphocytes (TILs), vascular invasion, and ulceration. The Mann-Whitney test was used for comparing two groups, whereas the Kruskal-Wallis test and Dunn’s multiple comparisons test were performed for the analyses of more than two groups [file 12967_2024_5622_MOESM4_ESM.jpg]
